# 5-Aminopyrazole as precursor in design and synthesis of fused pyrazoloazines

**DOI:** 10.3762/bjoc.14.15

**Published:** 2018-01-25

**Authors:** Ranjana Aggarwal, Suresh Kumar

**Affiliations:** 1 Department of Chemistry, Kurukshetra University, Kurukshetra-136119, Haryana, India

**Keywords:** 5-aminopyrazoles, fused pyrazole derivatives, pyrazolopyridines, pyrazolopyrimidines, pyrazolotriazines

## Abstract

The condensation of 5-aminopyrazole with various bielectrophilic moieties results in the formation of pyrazoloazines, an interesting array of fused heterocyclic systems. The development of new synthetic routes towards pyrazoloazines for their biological and medicinal exploration is an attractive area for researchers throughout the world. The present review focuses on various synthetic methods developed in the last decade for the synthesis of differently substituted pyrazoloazines by a broad range of organic reactions by means of 5-aminopyrazole as a precursor.

## Review

**Figure 1:**
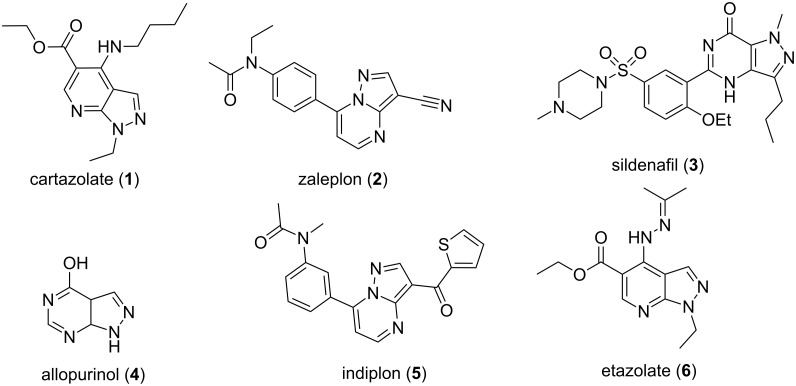
Selected examples of drugs with fused pyrazole rings.

Pyrazole and its derivatives are known to exhibit significant biological and pharmacological activities such as: anticancer [1–2], anti-inflammatory [3–4], antioxidant [5], antibacterial [6–8], analgesic [9], antiviral [10–11], antimicrobial [12–13], antifungal [6], antiglycemic [14], antiamoebic [15] and antidepressive [16–17]. Considering the immense biological properties pyrazole is one of the most widely studied nitrogen-containing heterocyclic nuclei. Fused pyrazole derivatives are composed of the pyrazole nucleus attached to other heterocyclic moieties which enable them to exhibit improved pharmacological activities compared to the isolated fragments. These compounds are currently used in several marketed drugs like cartazolate (**1**), zaleplon (**2**), sildenafil (**3**), allopurinol (**4**), indiplon (**5**), etazolate (**6**) etc. (Figure 1). Fused pyrazole derivatives, especially pyrazoloazines have been reported to mimic purine bases, present in DNA and RNA, due to close structural resemblance.

**Figure 2:**
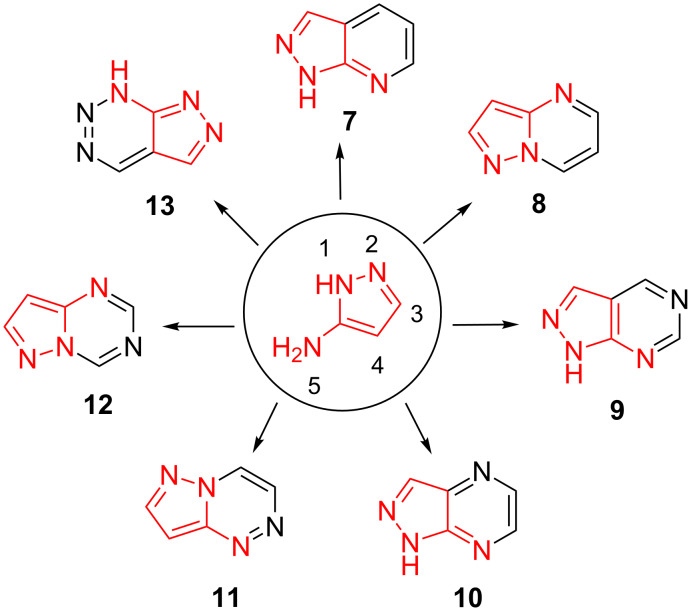
Typical structures of some fused pyrazoloazines from 5-aminopyrazoles.

In addition to the immense biological potential related to fused pyrazoles, their synthetic potential needs to be reviewed for further improvements and extension of interests. Various efforts have been developed for the synthesis of pyrazole-based fused heterocycles. 5-Aminopyrazoles have been extensively employed as useful synthons in designing and constructing a plethora of fused pyrazoloazines of potential synthetic and medicinal interest viz pyrazolo[3,4-*b*]pyridines **7** [18], pyrazolo[1,5-*a*]pyrimidines **8** [19], pyrazolo[3,4-*d*]pyrimidines **9** [20–21], pyrazolo[3,4-*b*]pyrazines **10** [22], pyrazolo[5,1-*c*]-1,2,4-triazines **11** [23], pyrazolo[1,5-*a*]-1,3,5-triazines **12** [24], pyrazolo[3,4-*d*][1,2,3]triazines **13** [25] (Figure 2).

A number of review articles have been published by us and others highlighting the synthetic and biological aspects of 5-aminopyrazoles [26–28] as well as on the synthesis of fused pyrazole derivatives [25]. However, a perusal of literature reveals that the importance of 5-aminopyrazoles as synthetic precursors for fused heterocycles has not been reported till now to the best of our knowledge. Recent literature shows resurgence of interest in the chemistry and bioactivity of 5-aminopyrazole derivatives leading to improvements in several already known reactions and syntheses of various fused heterocyclic derivatives with various biological activities. Considering the synthetic importance of 5-aminopyrazoles and synthesis of fused pyrazole derivatives with the need for a more general collection, herein we report an exhaustive overview of the main developments in the last decade in the chemistry of 5-aminopyrazoles for the design and synthesis of fused pyrazoloazines.

### The typical reactivity of 5-aminopyrazoles

5-Aminopyrazoles are polyfunctional compounds possessing three typical nucleophilic sites: 4-CH, 1-NH and 5-NH_2_ with the following reactivity order: 5-NH_2_ > 1-NH > 4-CH. These positions have been used to construct various fused heterocyclic rings where 5-aminopyrazoles undergo cyclization and cycloaddition on reaction with bielectrophiles. Due to the large number of references, reactions of 5-aminopyrazoles with various reagents to construct a six membered ring with pyrazole are discussed. The synthetic methods have been arranged in order of the ascending number of heteroatoms in the azine ring. The systematic arrangement in this review explores the possibility of providing practical guidance to synthetic chemists for further research.

### Synthesis of pyrazolo[3,4-*b*]pyridines

Pyrazolo[3,4-*b*]pyridines are important fused heterocycles due to their well-known synthetic and medicinal potential as good vasodilators [29], hypotensive [30], HIV reverse transcriptase inhibitors [31], protein kinase inhibitors [32], antiallergic [33], antioxidant [34] and as fungicide [35]. Also, the pyrazolo[3,4-*b*]pyridine ring system is a key structure in drug discovery and has become the main component in many medicinally important compounds. The large number of synthetic routes to pyrazolo[3,4-*b*]pyridines and their applications brings great interest in this area. The most commonly applied method for the preparation of pyrazolo[3,4-*b*]pyridines uses 5-aminopyrazole as synthetic precursor [36–39]. Regardless to substantial studies in this field, researchers are still focused to provide convenient regioselective synthetic methods with mild conditions and good yields of the reactions [40–41].

**Scheme 1:**
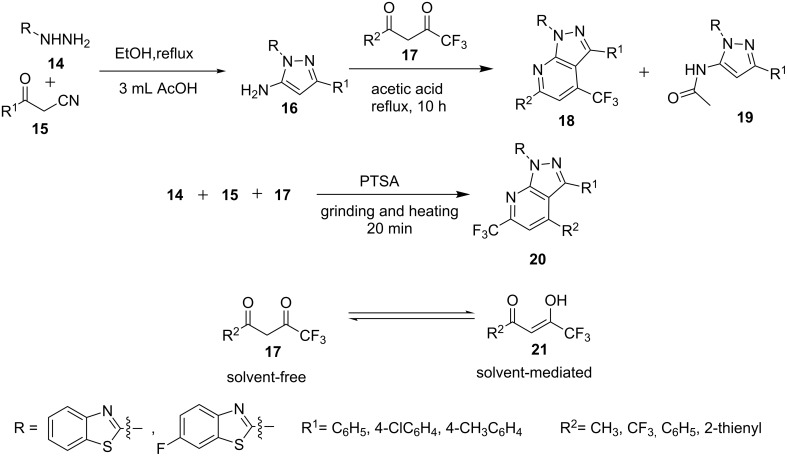
Regiospecific synthesis of 4 and 6-trifluoromethyl-1*H*-pyrazolo[3,4-*b*]pyridines.

Aggarwal et al. [42] reported the regiospecific synthesis of 4-trifluoromethyl-1*H*-pyrazolo[3,4-*b*]pyridines **18** by the reaction of 5-aminopyrazole **16** with trifluoromethyl-β-diketones **17** in refluxing acetic acid (Scheme 1). In the same report the other regioisomers 6-trifluoromethylpyrazolo[3,4-*b*]pyridines **20** were obtained under multicomponent solvent-free conditions by the reaction of hydrazine **14**, β-ketonitrile **15** and β-diketone **17** as an exclusive product. The structures of both the regioisomers have been confirmed unambiguously by HMBC, HMQC and ^19^F NMR studies. The authors proposed that trifluoromethyl-β-diketone exists mainly in keto form **17** under solvent-free conditions whereas under solvent-mediated conditions the enolic form **21** towards the carbonyl carbon that carries the CF_3_ group is predominant. The keto form **17** results in the formation of 6-trifluoromethylpyrazolo[3,4-*b*]pyridines **20** by attack of the 5-NH_2_ group (from 5-aminopyrazole **16**) on the more electrophilic carbonyl group attached to CF_3_ (from trifluoromethyl-β-diketones **17**) whereas the enolic form **21** reacts with the less nucleophilic C-4 of 5-aminopyrazole and leads to the formation of 4-CF_3_ product **18**. The formation of acetamide **19** as byproduct under solvent-mediated conditions was also observed due to the reaction of NH_2_ group with acetic acid.

**Scheme 2:**
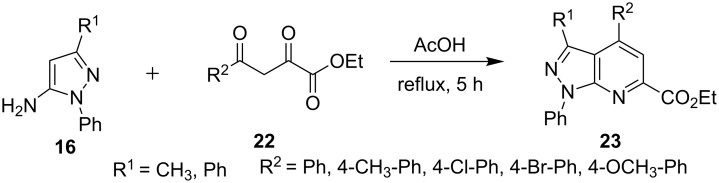
Synthesis of pyrazolo[3,4-*b*]pyridine-6-carboxylates.

Bardajee et al. [43] reported the synthesis of ethyl 1,3,4-triphenyl-1*H*-pyrazolo[3,4-*b*]pyridine-6-carboxylate (**23**) from the reaction of 5-aminopyrazole (R = Ph, **16**) and ethyl 2,4-dioxo-4-phenylbutanoate (**22**, Scheme 2). The presence of an electron-withdrawing group on the aryl ring provided higher yields due to the increased electrophilicity of the carbonyl carbon. Electron-donating groups on the contrary decreased the electrophilicity of the carbonyl carbon and hence resulted in lower yields.

**Scheme 3:**
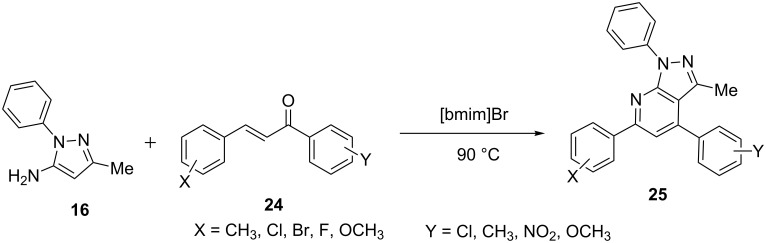
Synthesis of 1,4,6-triaryl-1*H*-pyrazolo[3,4-*b*]pyridines with ionic liquid .

The synthesis of 3-methyl-1,4,6-triaryl-1*H*-pyrazolo[3,4-*b*]pyridines **25** was described by Shi et al. [44] from the reaction of 3-methyl-1-phenyl-1*H*-pyrazol-5-amine (R = Ph, R^1 ^**=** Me, **16**) and α,β-unsaturated ketones **24** (Scheme 3) in the ionic solvent [bmim]Br at 90 °C with excellent yield. Variation of the aryl substituents on the α,β-unsaturated ketones **24** has no significant effect on the reaction. The reaction was proposed to occur through a sequence of Michael addition, cyclization, dehydration and aromatization reactions. The use of ionic liquids (non-volatile solvents) over toxic organic solvents makes it an environmentally benign process [45–46].

**Scheme 4:**
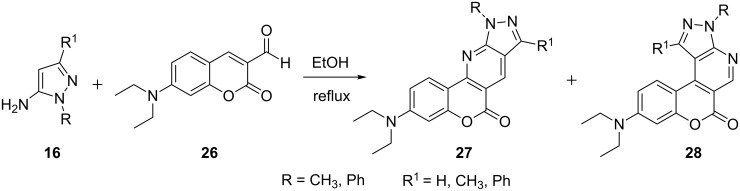
Synthesis of coumarin-based isomeric tetracyclic pyrazolo[3,4-*b*]pyridines.

The synthesis of isomeric tetracyclic pyrazolo[3,4-*b*]pyridine-based coumarin chromophores **27** and **28** was reported by Chen et al. [47] starting from 7-diethylaminocoumarin-3-aldehyde (**26**) and 5-aminopyrazole derivatives **16** (Scheme 4). The structure of the synthesized compounds was confirmed by X-ray crystallography, ^1^H and ^13^C NMR and HRMS studies. The relationships between the structures and chemical properties of these compounds were also investigated by techniques like fluorescence spectroscopy, single photon counting technique, cyclic voltammetry, thermogravimetric analysis, and DFT calculations.

**Scheme 5:**
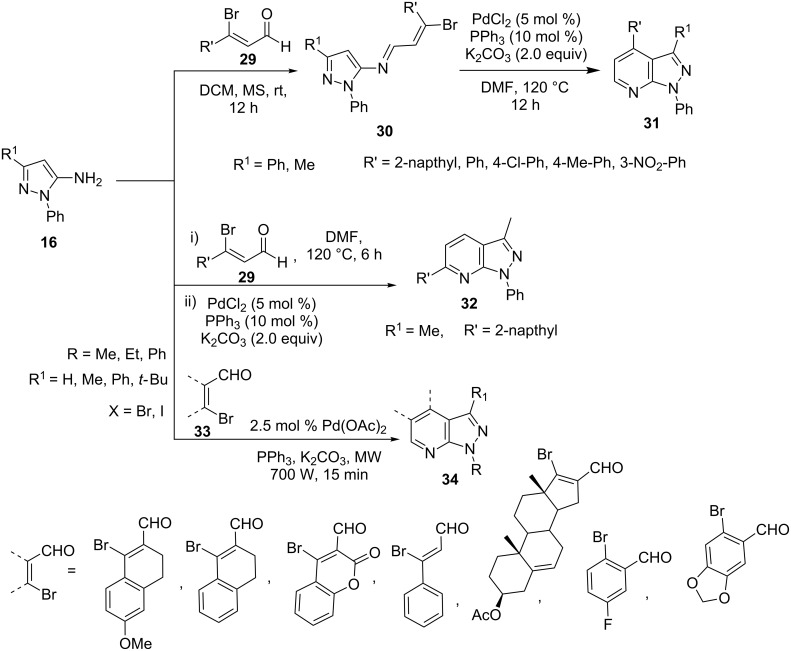
Synthesis of 6-substituted pyrazolo[3,4-*b*]pyridines under Heck conditions.

Boruah et al. [48–49] developed an efficient method for the construction of regioisomeric 1,3,4-trisubstituted pyrazolo[3,4-*b*]pyridines **32** and **34** (Scheme 5). In situ cyclocondensation of β-halovinyl aldehydes **29** with 5-aminopyrazoles (R = Ph, **16**) under Heck conditions in the presence of Pd(OAc)_2_ with xantphos (4,5-bis(diphenylphosphino)-9,9-dimethylxanthene) gave 6-substituted pyrazolo[3,4-*b*]pyridines **34**. On the other hand, isolated imine intermediate **30** under similar conditions provided 4-substituted pyrazolo[3,4-*b*]pyridine **31** in DMF (Scheme 5). This intramolecular coupling reaction provided highly efficient synthetic procedure for the design and synthesis of pyrazolo[3,4-*b*]pyridine-nucleus-based pharmacological agents with high regioselectivity.

Working on similar lines Boruah et al. [49] further explored the reactivity of 5-aminopyrazoles **16** with β-halovinyl/aryl aldehydes **33** under conventional heating and microwave conditions in DMF and DMSO with Pd(OAC)_2_ (2.5 mol %) catalyst with PPh_3_ as ligand (Scheme 5). Interestingly, high yields of the corresponding pyrazolo[3,4-*b*]pyridines **34** were obtained when reactions were carried under solvent-free microwave irradiation. The synthesized pyrazolo[3,4-*b*]pyridines have shown potential cytotoxic activity against cervical HeLa and prostate DU 205 cancer cell lines.

**Scheme 6:**
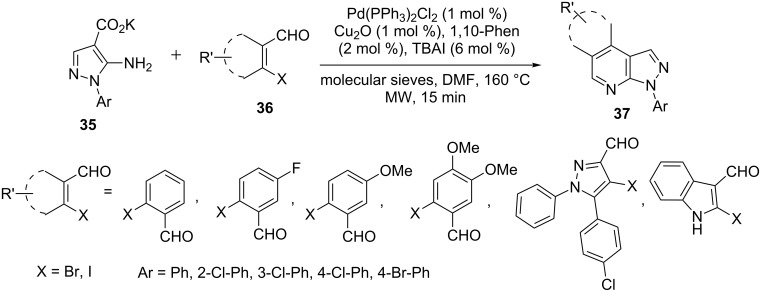
Microwave-assisted palladium-catalyzed synthesis of pyrazolo[3,4-*b*]pyridines.

A similar in situ intramolecular cyclization of 5-aminopyrazole-4-caroxylate **35** with β-haloaldehydes **36** via the corresponding imine derivative was carried out in presence of Pd(PPh_3_)_2_Cl_2_ (1.0 mol %), Cu_2_O (1.0 mol %), 1,10-phenanthroline (2.0 mol %), TBAI (6 mol %), by Batra et al. [50] to generate the pyrazolo[3,4-*b*]pyridine nucleus **37** (Scheme 6).

**Scheme 7:**
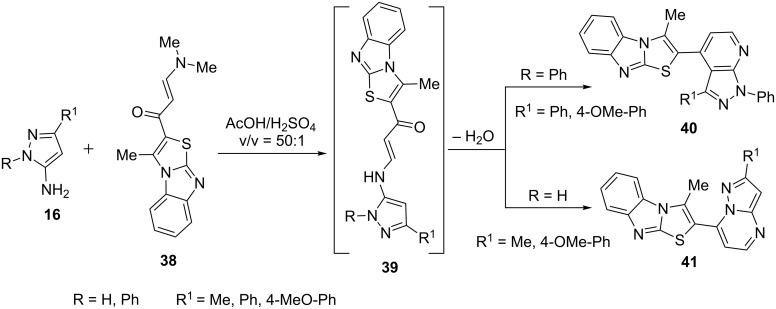
Acid-catalyzed synthesis of pyrazolo[3,4-*b*]pyridines via enaminones.

Aziz et al. [51] developed an acid-catalyzed synthesis of pyrazolo[3,4-*b*]pyridine derivatives **40** through the reaction of enaminone **38** with 5-aminopyrazole **(**R = Ph, **16)** in acetic acid (Scheme 7). The proposed reaction mechanism involves the generation of new enaminone intermediate **39** which underwent condensation and cyclization within C-4 of 5-aminopyrazole and the carbonyl group of the enaminone to generate pyrazolo[3,4-*b*]pyridine derivatives **40**. However, the formation of pyrazolo[1,5-*a*]pyrimidine **41**, a structural isomer of **40** was obtained when 1-NH-5-aminopyrazole (R = H, **16**) was condensed with **38**. It was attributed to cyclocondensation between 1-NH (5-aminopyrazole) and the carbonyl carbon of the enaminone. The compounds were found to have cytotoxicity against the normal fibroblast (BHK) cell line and antitumor activity against the colon cancer cell line CaCO-2.

**Scheme 8:**
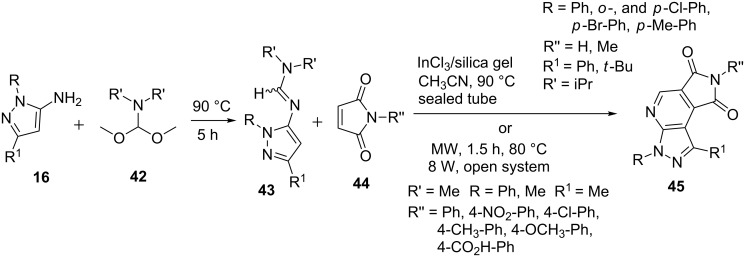
Synthesis of pyrazolo[3,4-*b*]pyridines via aza-Diels–Alder reaction.

Lin et al. [52] developed the synthesis of pyrazolo[3,4-*b*]pyridine derivatives **45** via aza-Diels–Alder reaction of pyrazolylimines **43** with maleimides **44** (Scheme 8). Pyrazolylimines **43** were in turn obtained from the reaction of 5-aminopyrazole **16** with diisopropylformamide dimethyl acetal (R’ = isopropyl, **42**). The reactions were carried out with various metal catalysts in acetic acid and acetonitrile solvents but reactions carried in acetic acid in presence of silica gel impregnated with indium trichloride provided the best results. Júnior et al. [53] also used *N*,*N*-dimethylpyrazoylimines **42** with *N*-arylmaleimides **44** in a solvent-free methodology based on microwave-assisted (80 W, 80 °C, 1.5 h) hetero-Diels–Alder reaction for the synthesis of pyrazolo[3,4-*b*]pyridine derivatives **45** (Scheme 8).

**Scheme 9:**
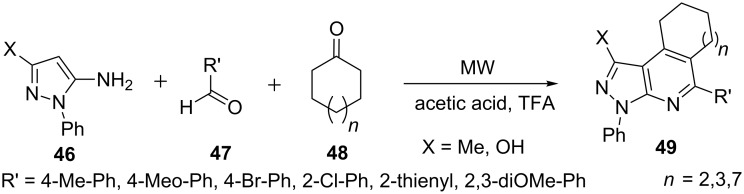
Synthesis of macrocyclane fused pyrazolo[3,4-*b*]pyridine derivatives.

Jiang et al. [54] described the synthesis of macrocyclane-fused pyrazolo[3,4-*b*]pyridine derivatives **49** by the reaction of 5-aminopyrazole derivative **46**, arylaldehydes **47** and cyclic ketones **48** in various solvents like acetonitrile, ethylene glycol, acetic acid, DMF under MW conditions at 80 °C (Scheme 9). The best results (72–80% yields) were obtained by carrying out the reaction in acetic acid with the addition of TFA as promoter at 80 °C to 140 °C.

**Scheme 10:**
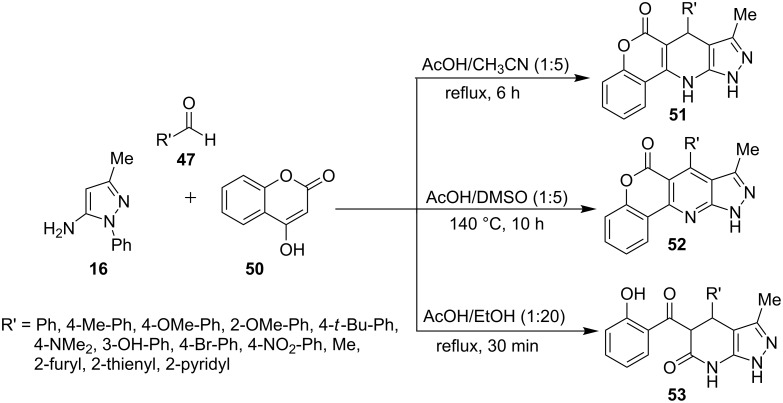
Three-component synthesis of 4,7-dihydro-1*H*-pyrazolo[3,4-*b*]pyridine derivatives.

A three-component reaction of 5-aminopyrazole **16**, 4-hydroxycoumarin (**50**) and aldehydes **47** was studied by Liu et al. [55] in various solvents like acetonitrile, dichloromethane, toluene and DMSO in the presence of catalysts like ZrCl_4_, InCl_3_, FeCl_3_, L-proline etc. (Scheme 10). Whereas the reaction in acetic acid/acetonitrile (1:5) provided 4,7-dihydro-1*H*-pyrazolo[3,4-*b*]pyridine derivatives **51**, dimethyl sulfoxide/acetic acid (5:1) yielded the corresponding aromatized pyrazolo[3,4-*b*]pyridine derivatives **52** exclusively. In acetic acid/ethanol combination an unexpected product 4,5-dihydro-1*H*-pyrazolo[3,4-*b*]pyridine-6(7*H*)-one **53** was formed due to C–O bond cleavage from cyclic ester **51**.

**Scheme 11:**
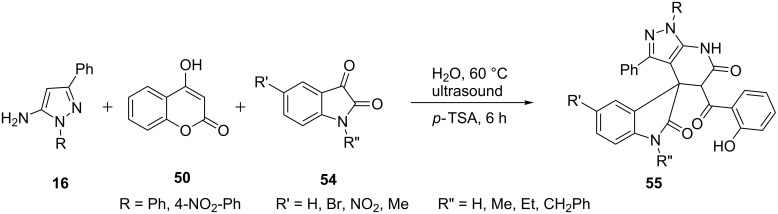
Ultrasonicated synthesis of spiro[indoline-3,4'-pyrazolo[3,4-*b*]pyridine]-2,6'(1'*H*)-diones.

Bazgir et al. [56] described the synthesis of spiro[indoline-3,4'-pyrazolo[3,4-*b*]pyridine]-2,6'(1'*H*)-diones **55** by an efficient three-component procedure from the reaction of 5-aminopyrazole **16** and 4-hydroxycoumarin (**50**) with isatin **54** under ultrasound irradiation in water (Scheme 11). Solvent and catalytic screening for the reaction have shown that water in presence of *p*-TSA at 60 °C on heating for 6 hours provide best results with excellent yields.

**Scheme 12:**
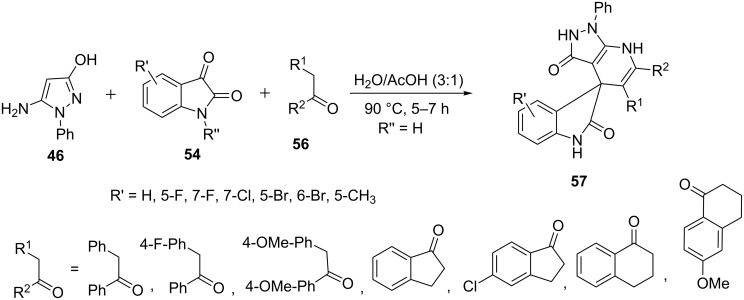
Synthesis of spiro[indoline-3,4'-pyrazolo[3,4-*b*]pyridine] derivatives under conventional heating conditions.

Recently, Wang et al. [57] also described the construction of spiro[indoline-3,4'-pyrazolo[3,4-*b*]pyridine] derivatives **57** from the multicomponent reaction of 5-amino-3-hydroxy-1-phenyl-1*H-*pyrazole (**46**), ketones **56** and isatin **54** in water/acetic acid (3:1) at 90 °C (Scheme 12).

**Scheme 13:**
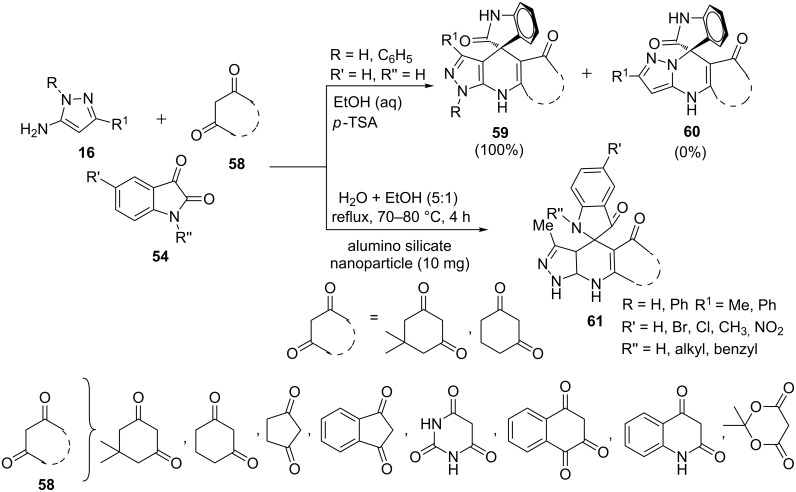
Nanoparticle-catalyzed synthesis of pyrazolo[3,4-*b*]pyridine-spiroindolinones.

Quiroga et al. [58] reported the synthesis of the pyrazolo[3,4-*b*]pyridine-spiroindolinone nucleus **59** with a high degree of regioselectivity without formation of the regioisomeric pyrazolo[1,5-*a*]pyrimidine **60** involving three-component reaction of 5-aminopyrazole **16**, isatin **54** and cyclic β-diketones **58** in aqueous ethanol with *p*-TSA as catalyst (Scheme 13). Bhaumik et al. [59] carried out a similar reaction of 5-aminopyrazole (R = H, R^1^** =** Me, **16**), isatin **54** and cyclic-1,3-diones **58** in aqueous ethanol using aluminosilicate nanoparticles catalyst to yield pyrazolo[3,4-*b*]pyridines **61** (Scheme 13).

**Scheme 14:**
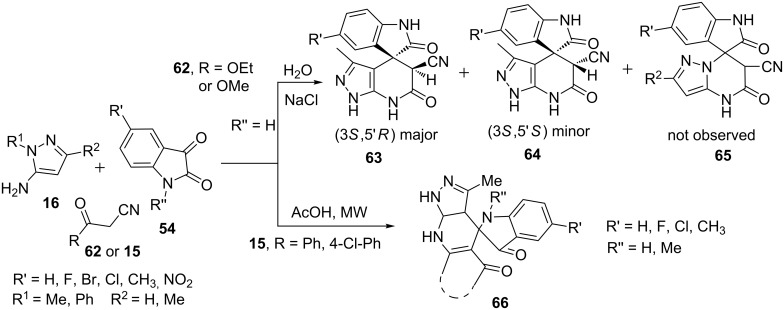
Microwave-assisted multicomponent synthesis of spiropyrazolo[3,4-*b*]pyridines.

Dandia et al. [60] carried out the multicomponent synthesis of spiropyrazolo[3,4-*b*]pyridines **63** and **64** starting from 5-aminopyrazole (R = H, R^1^** =** Me, **16**), isatin **54** and α-cyanoacetic ester **62** or **15** in aqueous-mediated reaction in presence of NaCl. Regioisomeric pyrazolo[1,5-*a*]pyrimidines **65** were not formed in any of the tried reaction conditions. An increase in the amount of NaCl from 2.5 to 10 mol % resulted in gradual increase of the yield of the desired product **63** from 85% to 89% and 93%, respectively (Scheme 14). Recently, Jiang et al. [61] have also developed a microwave-assisted synthesis of spiropyrazolo[3,4-*b*]pyridines **66** via a similar type of three-component reaction of 5-aminopyrazole **16**, isatin **54** and 3-oxo-3-phenylpropanenitriles **15** in acetic acid under microwave irradiation at 80 °C in just 20 minutes (Scheme 14).

**Scheme 15:**
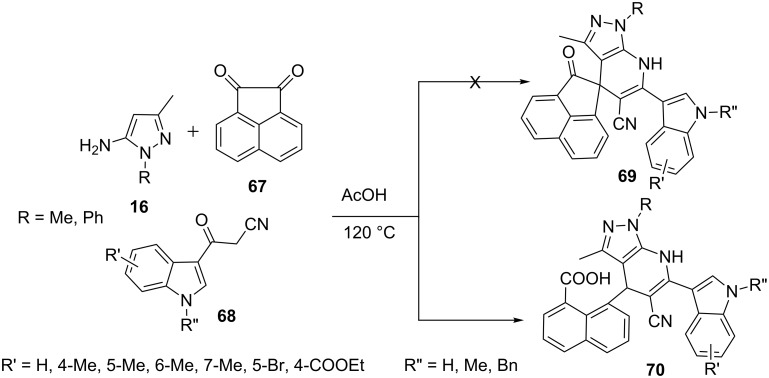
Unexpected synthesis of naphthoic acid-substituted pyrazolo[3,4-*b*]pyridines.

Hao et al. [62] described the unexpected synthesis of naphthoic acid substituted pyrazolo[3,4-*b*]pyridine derivatives **70** via a three-component reaction of 5-aminopyrazole (R = Me, **16**) with acenaphthenequinone **67** and β-ketonitrile derivative **68** in glacial acetic acid instead of expected spiropyrazolo[3,4-*b*]pyridines **69** (Scheme 15). The structures of the products were confirmed by spectral and X-ray crystallographic data. This method provides the first direct conversion of acenaphthenequinone to a naphthoic acid fragment via C–C bond cleavage in a single step.

**Scheme 16:**
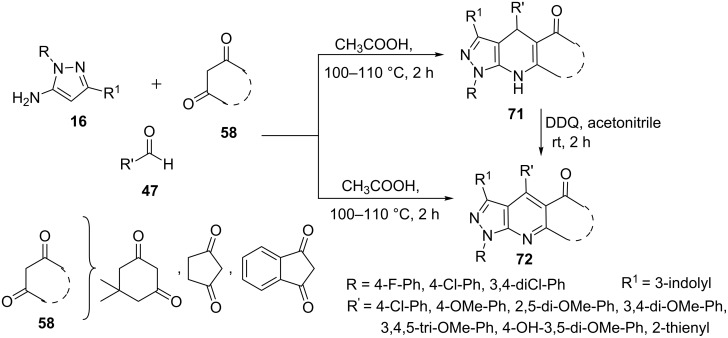
Multicomponent synthesis of variously substituted pyrazolo[3,4-*b*]pyridine derivatives.

Recently, D. Anand et al. [63] have reported the synthesis of pyrazolo[3,4-*b*]pyridine derivatives **71** and **72** through the multicomponent reaction of 1-aryl-3-indolyl-5-aminopyrazoles **16**, cyclic β-diketones **58** and aryl aldehydes **47** (Scheme 16). The reaction resulted in good yields of pyrazolo[3,4-*b*]pyridines **72** but in few cases 4,7-dihydropyrazolo[3,4-*b*]pyridines **71** were formed as major product even after prolonged heating. 4,7-Dihydropyrazolo[3,4-*b*]pyridines **71** were dehydrogenated to their aromatic counterparts **72** in presence of 2,3-dichloro-5,6-dicyanobenzoquinone (DDQ) in acetonitrile.

**Scheme 17:**
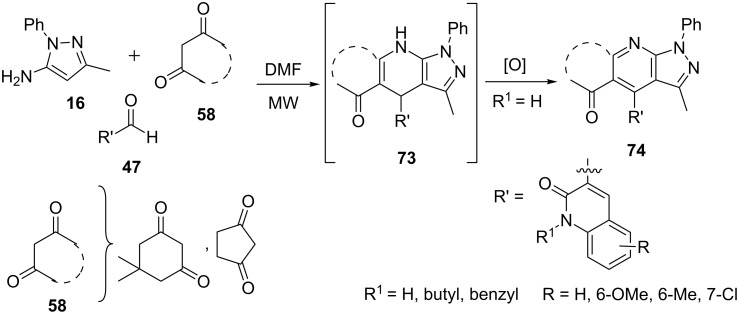
Three-component synthesis of 4,7-dihydropyrazolo[3,4-*b*]pyridines and pyrazolo[3,4-*b*]pyridines.

Insuasty et al. [64] adapted a similar synthetic strategy for the construction of 4,7-dihydropyrazolo[3,4-*b*]pyridines **73** and pyrazolo[3,4-*b*]pyridines **74** by a three-component reaction of 5-aminopyrazoles **16**, cyclic β-diketones **58** and heteroaryl aldehydes **47** (Scheme 17). The reaction under conventional heating in DMF provided best results with high yields of the corresponding pyrazolo[3,4-*b*]pyridines **74**.

**Scheme 18:**
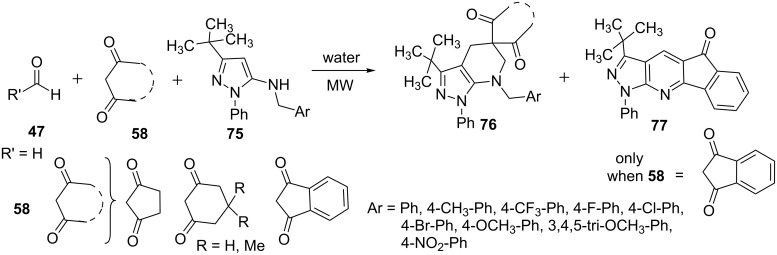
Synthesis of pyrazolo[3,4-*b*]pyridine-5-spirocycloalkanediones.

The multicomponent reactions of 5-(4-substituted-benzylamino)pyrazoles **75**, cyclic-β-diketones **58** and formaldehyde (R’= H, **47**) were performed under microwave and conventional heating conditions by Quiroga et al. [65] (Scheme 18). Both the reaction conditions resulted in the formation of pyrazolo[3,4-*b*]pyridine-5-spirocycloalkanediones **76** but an additional compound 3-*tert*-butyl-1-phenylindeno[2,3-*e*]pyrazolo[3,4-*b*]pyridine **77** was formed in the reaction when indandione **58** was used as β-diketone which was attributed to the loss of the benzyl fragment from 5-aminopyrazole derivative **75**. Microwave-assisted reactions went to completion in very short time (5 min) compared to reactions under conventional heating conditions (24 hours).

**Scheme 19:**
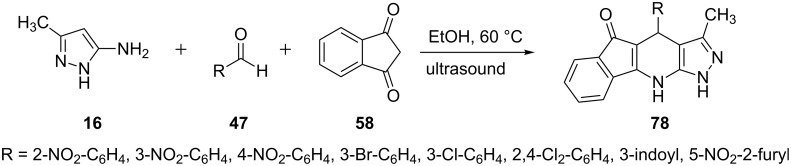
Ultrasound-mediated three-component synthesis of pyrazolo[3,4-*b*]pyridines.

A three-component reaction of 5-aminopyrazole **16**, arylaldehydes **47** and indandione **58** under ultrasonic irradiation in ethanol was developed by Nikpassand et al. [66] to synthesize pyrazolo[3,4-*b*]pyridine derivatives **78** (Scheme 19). Ultrasound-mediated reactions yielded the corresponding pyrazolo[3,4-*b*]pyridine derivatives **78** in 4–5 minutes with 88–97% yields.

**Scheme 20:**
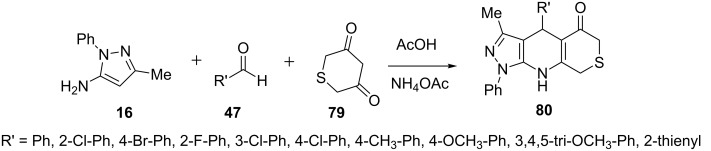
Multicomponent synthesis of 4-aryl-3-methyl-1-phenyl-4,6,8,9-tetrahydropyrazolo [3,4-*b*]thiopyrano[4,3-*e*]pyridin-5(1*H*)-ones.

Yao et al. [67] demonstrated that 4-aryl-3-methyl-1-phenyl-4,6,8,9-tetrahydropyrazolo[3,4-*b*]thiopyrano[4,3-*e*]pyridin-5(1*H*)-one derivatives **80** could be synthesized from a three-component reaction of 5-aminopyrazole **16**, arylaldehyde **47**, and 2*H*-thiopyran-3,5(4*H*,6*H*)-dione (**79**) in glacial acetic acid in presence of ammonium acetate (Scheme 20).

**Scheme 21:**
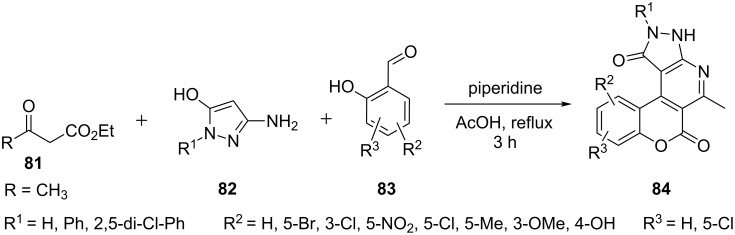
Synthesis of 2,3-dihydrochromeno[4,3-*d*]pyrazolo[3,4-*b*]pyridine-1,6-diones.

The multicomponent reaction of 5-amino-3-hydroxypyrazoles **82**, substituted salicylic aldehydes **83** and acetylacetic ester **81** in acetic acid with few drops of piperidine was reported to give 2,3-dihydrochromeno[4,3-*d*]pyrazolo[3,4-*b*]pyridine-1,6-diones **84** by Frolova et al. [68] (Scheme 21). The reactions with ethyl benzoylacetate as ketoester component had not provided the corresponding pyrazolo[3,4-*b*]pyridines which was attributed to the change in the electronic and steric environments. All the synthesized compounds were reported as good antimicrobial agents.

**Scheme 22:**
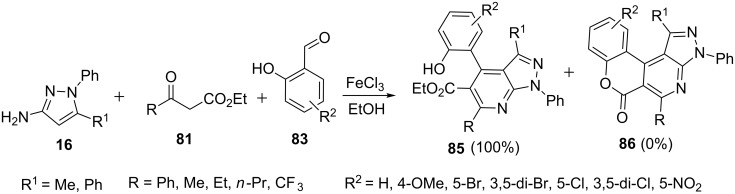
FeCl_3_-catalyzed synthesis of *o*-hydroxyphenylpyrazolo[3,4-*b*]pyridine derivatives.

Recently the reaction of β-ketoesters **81** as in the three-component reaction with 5-aminopyrazoles **16** and substituted salicylic aldehydes **83** was also studied by Fan et al. [69]*.* An extensive survey of catalysts and solvents identified 0.2 equivalents of FeCl_3_ and ethanol as optimal catalyst and solvent, respectively, with which *o*-hydroxyphenylpyrazolo[3,4-*b*]pyridine derivatives **85** were obtained in 89% yields with no formation of the cyclized isomer chromenopyrazolo[3,4-*b*]pyridine **86**. The reaction in the presence of other catalysts like L-proline, InCl_3_ and ZrCl_4_ also resulted in the formation of *o*-hydroxyphenylpyrazolo[3,4-*b*]pyridine derivatives **85** but no product was formed in iodine- and acetic acid-catalyzed reactions (Scheme 22).

**Scheme 23:**
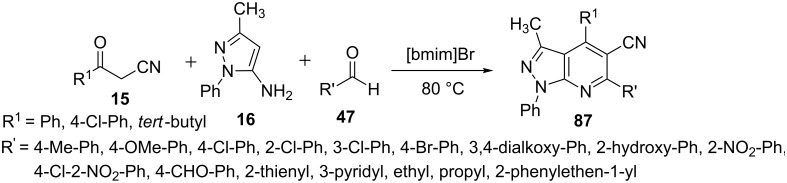
Ionic liquid-mediated synthesis of pyrazolo[3,4-*b*]pyridines.

Huang et al. [70] investigated a three-component reaction of β-ketonitriles **15**, 5-aminopyrazole **16** and aldehydes **47** in various organic solvents and ionic liquids to synthesize pyrazolo[3,4-*b*]pyridine derivative **87** (Scheme 23). Ionic liquids provided high yields of **87** in very short time with the best results obtained in [bmim]Br whereas organic solvents resulted in low yields and took longer time for the completion of reaction.

**Scheme 24:**
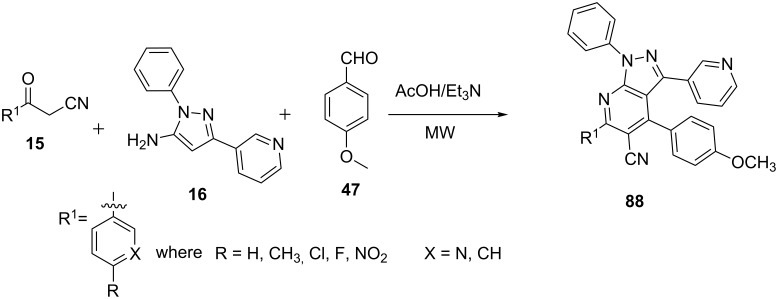
Microwave-assisted synthesis of pyrazolo[3,4-*b*]pyridines.

El-borai et al. [71] accomplished the synthesis of pyrazolo[3,4-*b*]pyridine derivatives **88** in which the multicomponent reactions of β-ketonitriles **15**, 5-aminopyrazole **16** and anisaldehyde (**47**) were carried out in acetic acid under conventional heating and microwave assistance (Scheme 24). The microwave-assisted reaction provided better yields of pyrazolo[3,4-*b*]pyridine derivatives **88** as compared to reactions under conventional heating conditions in short time.

**Scheme 25:**
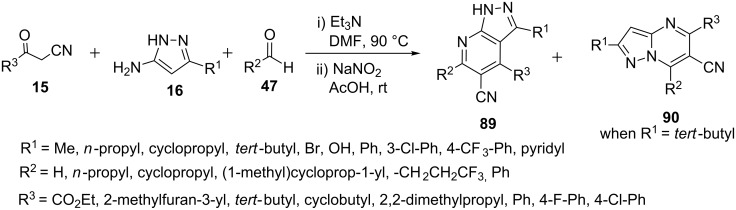
Multicomponent synthesis of pyrazolo[3,4-*b*]pyridine-5-carbonitriles.

Hill et al. [72–73] reported the synthesis of pyrazolo[3,4-*b*]pyridines **89** from the reaction β-ketonitriles **15** with 5-aminopyrazole **16** and aldehydes **47** (1 equiv each) in presence of triethylamine (2 equiv) by heating the reaction mixture at 90 °C in DMF for 16 hours followed by treatment with sodium nitrite (3 equiv) in acetic acid at ambient temperature. In addition, when the R^1^ group has significant bulk (R^1^ = *tert*-butyl) the reaction results in the formation of pyrazolo[1,5-*a*]pyrimidine derivative **90** as an additional product. The authors proposed that the bulky group had significantly slowed down the rate of electrophilic aromatic substitution at C-4 on 1*H*-pyrazol-5-amine due to which the aza-Michael addition becomes competitive at N-1 which ultimately provides pyrazolo[1,5-*a*]pyrimidine derivative **90** as additional product (Scheme 25). The synthesized pyrazolo[3,4-*b*]pyridines **89** were found to be good mGluR5 positive allosteric modulators (PAMs) and therefore can be used to develop antipsychotic drugs to treat schizophrenia.

**Scheme 26:**
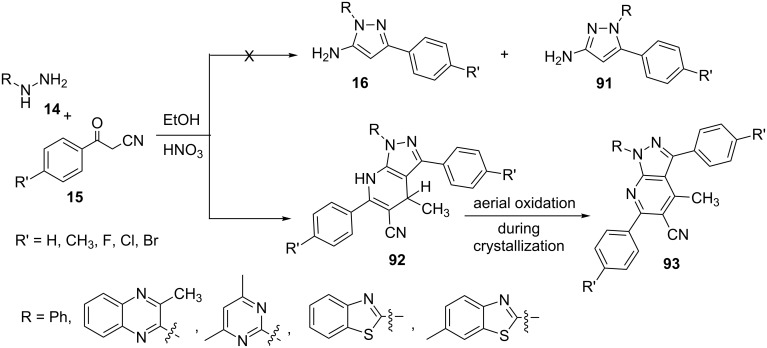
Unusual domino synthesis of 4,7-dihydropyrazolo[3,4-*b*]pyridine-5-nitriles.

In an interesting report Aggarwal et al. [74] described the synthesis of 4,7-dihydropyrazolo[3,4-*b*]pyridine-5-nitriles **92** from the reaction of β-ketonitriles **15** with several aryl/heteroaryl hydrazines **14** in ethanol with a catalytic amount of conc. HNO_3_ (Scheme 26). The authors carried out the reaction under acidic conditions expecting the formation of the regioisomeric 3/5-aminopyrazoles **16**/**91** but the reaction under the influence of conc. HNO_3_ resulted in the formation of an unexpected product which was characterized as 4,7-dihydropyrazolo[3,4-*b*]pyridine **92** through rigorous spectroscopic studies. However, X-ray crystallographic studies indicated that the 4,7-dihydropyrazolo[3,4-*b*]pyridine-5-nitriles **92** underwent aerial oxidation to its aromatic counterpart pyrazolo[3,4-*b*]pyridine **93** during crystallization and is propeller in shape. Additionally, non-planar rings due to propeller shape of compound **93** makes it chiral in nature. It was proposed that there is in situ oxidation of ethanol to ethanal by conc. HNO_3_ which turned the reaction into a multi-component domino assembly of reactants hydrazine **14**, β-ketonitriles **15** and acetaldehyde.

**Scheme 27:**
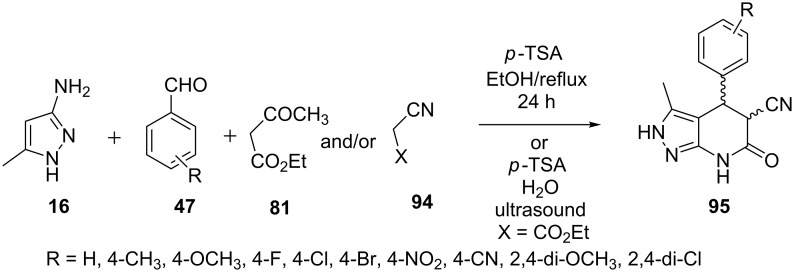
Synthesis of 4,5,6,7-tetrahydro-4*H*-pyrazolo[3,4-*b*]pyridines under conventional heating and ultrasound irradiation.

Rahmati [75] carried out a reaction of 5-aminopyrazole **16** with aldehydes **47** and ethyl cyanoacetate (**94**) in ethanol in presence of *p*-toluenesulfonic acid which resulted in a diastereomeric mixture of *cis*- and *trans*-4,5,6,7-tetrahydro-2*H*-pyrazolo[3,4-*b*]pyridines **95**. Benzaldehydes **47** with electron withdrawing groups provided better yields of the *cis*-isomer in slightly higher amounts than the *trans*-isomer. A four-component reaction having ethyl acetoacetate (**81**) as fourth component resulted in the formation of the same pyrazolo[3,4-*b*]pyridine derivative **95** showing no involvement of any additional fourth component (Scheme 27).

Dandia et al. [76] also reported a similar reaction of 5-aminopyrazole **16**, arylaldehyde **47** with ethyl cyanoacetate (**94**) under ultrasound irradiation in presence of *p*-TSA in water for the synthesis of 3-methyl-6-oxo-4-aryl-4,5,6,7-tetrahydro-4*H*-pyrazolo[3,4-*b*]pyridine-5-carbonitrile derivatives **95** (Scheme 27). All the synthesized compounds were tested for their effect on corrosion of mild steel (MS) in 1.0 M HCl with various experimental techniques like weight loss, electrochemical impedance spectroscopy (EIS), and potentiodynamic polarization techniques.

**Scheme 28:**
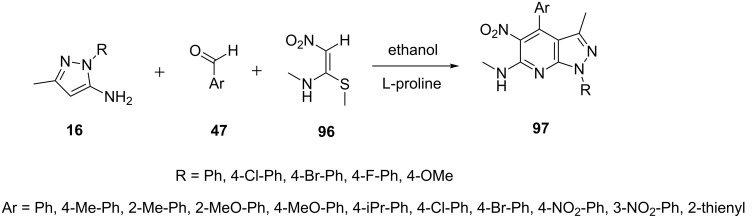
L-Proline-catalyzed synthesis of of pyrazolo[3,4-*b*]pyridine.

A three-component reaction of 5-aminopyrazole **16**, arylaldehyde **47** and *N*-methyl-1-(methylthio)-2-nitroethenamine (**96**) was studied by Gunasekaran et al. [77] (Scheme 28) in ethanol in presence of 30 mol % L-proline as catalyst at 78 °C which resulted in the production of pyrazolo[3,4-*b*]pyridine derivatives **97** in excellent yields.

**Scheme 29:**
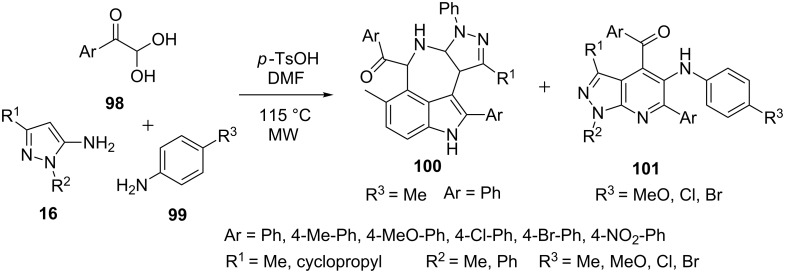
Microwave-assisted synthesis of 5-aminoarylpyrazolo[3,4-*b*]pyridines.

Jiang et al. [78] have investigated a microwave-irradiated reaction of 5-aminopyrazoles **16**, 2,2-dihydroxy-1-phenylethanone (**98**) and *p*-toluidine (**99**) under various polar and non-polar solvents with bronsted and lewis acid catalysts (Scheme 29). The reaction in dimethylformamide in presence of *p*-TSA resulted in the formation of azepino[5,4,3-*cd*]indole **100** instead of expected pyrazolo[3,4-*b*]pyridine derivatives **101**. However, the reactions of arylglyoxal **98** having an electron-donating group at C-4 position of the phenyl ring resulted in the formation of the desired pyrazolo[3,4-*b*]pyridines **101**.

**Scheme 30:**
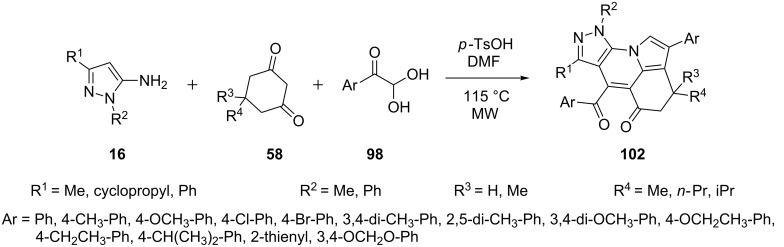
Microwave-assisted multi-component synthesis of pyrazolo[3,4-*e*]indolizines.

Wang et al. [79] studied the base-catalyzed multicomponent domino reaction of 5-aminopyrazoles **16**, cyclic 1,3-diones **58** and arylglyoxals **98** under microwave irradiation. Triethylamine (20 mol %) as base and DMSO as solvent at 120 °C provided best results with high yields of pyrazolo[3,4-*e*]indolizines (a derivative of pyrazolo[3,4-*b*]pyridines) **102** (Scheme 30).

### Synthesis of pyrazolo[1,5-*a*]pyrimidines

Pyrazolo[1,5-*a*]pyrimidines, structural isomers of pyrazolo[3,4-*b*]pyridines, are of interest because they constitute an important class of heterocycles which display biological and pharmacological activities and are useful precursors in the synthesis of many biologically active compounds [80–83]. Consequently, there has been an ongoing interest in the synthesis of pyrazolo[1,5-*a*]pyrimidines [84–86].

**Scheme 31:**
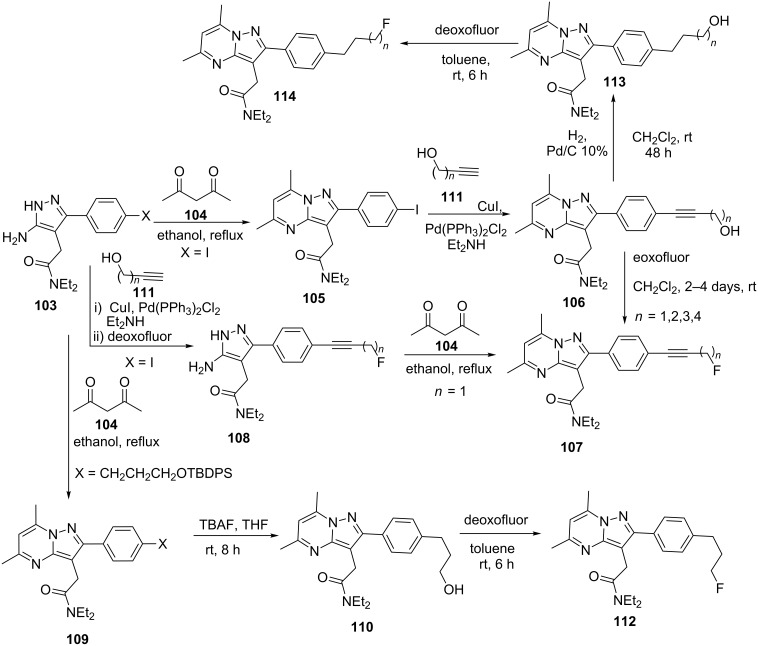
Synthesis of fluoropropynyl and fluoroalkyl substituted pyrazolo[1,5-*a*]pyrimidine.

Navarrete et al. [87] reported the reaction of acetylacetone (**104**) with 5-amino-3-(4-iodophenyl)pyrazole **103** in ethanol that gives pyrazolo[1,5-*a*]pyrimidine derivatives **105** which were subsequently used to prepare alkynyl alcohol **111** derivatives of pyrazolo[1,5-*a*]pyrimidines **106** by a Sonogashira coupling in 69–94% yields. Fluorodeoxygention of **106** using deoxofluor afforded fluoropropynyl-substituted pyrazolo[1,5-*a*]pyrimidine **107** with variable efficiency in terms of yields. Alternatively, by shuffling the steps of acetylacetone condensation and fluoroalkynylation, via 5-amino-3-(4-(fluoroalkynyl)phenyl)pyrazole **108** intermediate, a better and efficient route to synthesize **107** was developed (Scheme 31). Recently, the same research group [19] also reported the synthesis of fluoroalkyl-substituted pyrazolo[1,5-*a*]pyrimidine derivatives **112** and **114** using similar synthetic strategies (Scheme 31). Alkynyl alcohol derivatives of pyrazolo[1,5-*a*]pyrimidines **106** were hydrogenated to give alkyl alcohol-substituted pyrazolo[1,5-*a*]pyrimidines **113** which on treatment with deoxofluor resulted in the formation of fluoroalkyl-pyrazolo[1,5-*a*]pyrimidines **114**. Additionally, reaction of **104** with **103** in refluxing ethanol resulted in the formation of pyrazolo[1,5-*a*]pyrimidines **109** which on treatment with TBAF provided alkyl alcohol derivatives of pyrazolo[1,5-*a*]pyrimidines **110** which were later on converted to fluoroalkyl pyrazolo[1,5-*a*]pyrimidines **112** by treatment with deoxofluor.

**Scheme 32:**
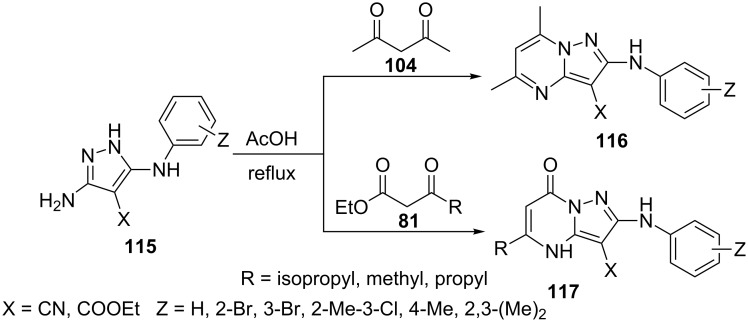
Acid-catalyzed synthesis of pyrazolo[1,5-*a*]pyrimidine derivatives.

Marjani et al. [88] have described the synthesis of pyrazolo[1,5-*a*]pyrimidine **116** and 4,7-dihydropyrazolo[1,5-*a*]pyrimidinone derivatives **117** by condensing 4-cyano/carboxylate-5-aminopyrazole derivatives **115** with acetylacetone (**104**) and various β-ketoesters **81**, respectively, in refluxing acetic acid with catalytic amount of suphuric acid (Scheme 32).

**Scheme 33:**
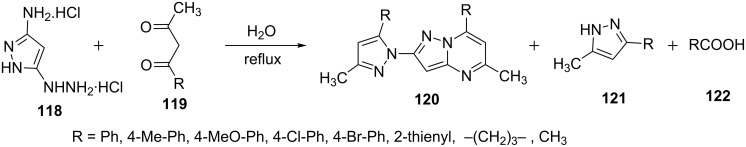
Chemoselective and regiospecific synthesis of 2-(3-methylpyrazol-1’-yl)-5-methylpyrazolo[1,5-*a*]pyrimidines.

The reaction of 3(5)-amino-5(3)-hydrazinopyrazole dihydrochloride (**118**) with symmetrical and unsymmetrical diketones **119** was studied by Aggarwal et al. [80,89] under aqueous conditions. The reaction exhibited a high level of chemoselectivity and regiospecificity yielding 2-(3-methylpyrazol-1-yl)-5-methylpyrazolo[1,5-*a*]pyrimidines **120** out of the four possible isomers (Scheme 33). In the case of arylbutadiones, formation of two more products in small amounts namely 3(5)-methyl-5(3)-phenyl-1*H*-pyrazole (**121**) obtained by CN bond cleavage and benzoic acid (**122**) was also observed (Scheme 33). The structure of the regioisomer was established unequivocally by performing ^1^H,^13^C-HMQC, ^1^H,^13^C- and ^1^H,^15^N-HMBC experiments. Aqueous mediated conditions makes it a sought after the procedure for the synthesis of pyrazolo[1,5-*a*]pyrimidines.

**Scheme 34:**
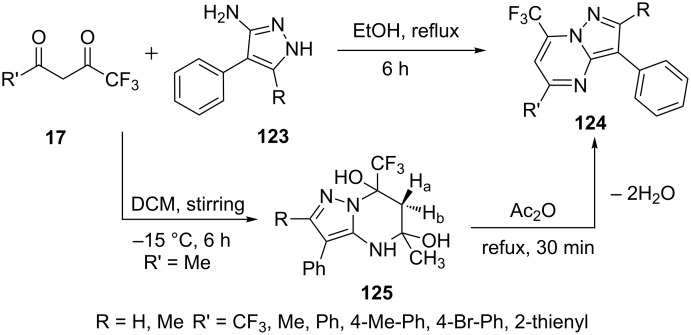
Regioselective synthesis of 7-trifluoromethylpyrazolo[1,5-*a*]pyrimidines.

In another report, Aggarwal et al. [90] have described a regioselective synthesis of 2-*H*/methyl-3-phenyl-7-trifluoromethylpyrazolo[1,5-*a*]pyrimidines **124** by condensing 4-aryl-5-aminopyrazoles **123** with an equimolar amount of trifluoromethyl-β-diketones **17**. To gain an insight of the reaction mechanism, the intermediate, 5-methyl-3-phenyl-7-trifluoromethyl-4,5,6,7-tetrahydropyrazolo[1,5-*a*]pyrimidine-5,7-diol **125** was isolated by performing the reaction in DCM at −15 °C for 6 h which was later converted to 7-trifluoromethylpyrazolo[1,5-*a*]pyrimidine derivative **124** by dehydration on refluxing with acetic anhydride (Scheme 34). All the synthesized compounds were screened for their anti-inflammatory activity.

**Scheme 35:**
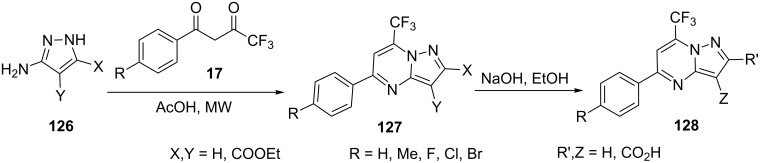
Microwave-assisted synthesis of 7-trifluoromethylpyrazolo[1,5-*a*]pyrimidine carboxylates.

Mulakayala and co-workers [91] synthesized 7-trifluoromethylpyrazolo[1,5-*a*]pyrimidine carboxylates **127** by the reaction of 5-aminopyrazole-4/3-ethylcarboxylates **126** with trifluoromethyl-β-diketones **17** in acetic acid under microwave heating, which were subsequently hydrolyzed to the corresponding pyrazolo[1,5-*a*]pyrimidine carboxylic acids **128** by treating with sodium hydroxide in ethanol at 65 °C. The compounds were screened for their cytotoxic activity against human colon carcinoma (Colo-205) cell lines (Scheme 35).

**Scheme 36:**
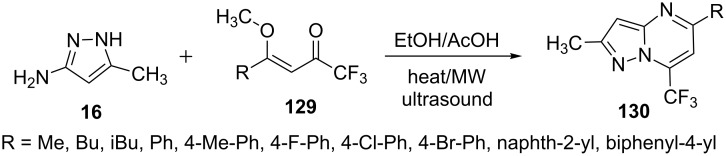
Microwave and ultrasound-assisted synthesis of 7-trifluoromethylpyrazolo[1,5-*a*]pyrimidines.

Buriol et al. [92] described the reaction of 5-aminopyrazole **16** with 4-alkoxy-1,1,1-trifluoro-3-alken-2-ones **129** to yield pyrazolo[1,5-*a*]pyrimidine derivatives **130** in acetic acid and ethanol using conventional heating, ultrasound and microwave conditions (Scheme 36). The reaction in ethanol provided best results with high yields of pyrazolo[1,5-*a*]pyrimidines **130**. The effect of microwave irradiation was found to be as efficient as of ultrasound radiations with better yields and shorter reaction times than conventional heating methods.

**Scheme 37:**
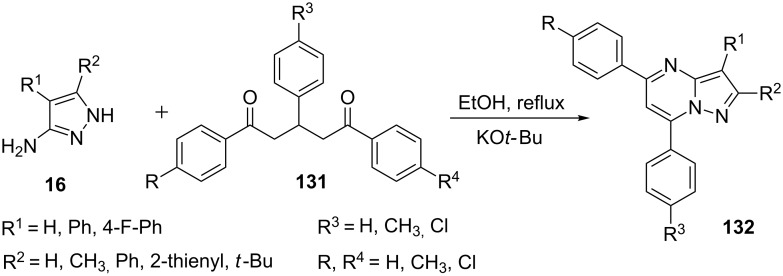
Base-catalyzed unprecedented synthesis of pyrazolo[1,5-*a*]pyrimidines via C–C bond cleavage.

Recently, Boruah et al. [93] reported an unprecedented synthesis of pyrazolo[1,5-*a*]pyrimidines **132** involving a C–C bond cleavage through KO*t*-Bu-catalyzed condensation of 1,3,5-trisubstituted pentane-1,5-diones **131** with substituted 5-aminopyrazoles **16** in ethanol. Symmetrical 1,5-dicarbonyls reacted efficiently with 5-aminopyrazoles **16** to give the corresponding substituted pyrazolo[1,5-*a*]pyrimidines **132** (Scheme 37). Moreover, the reaction of 1,5-dicarbonyls **131** with 5-amino-3-methylpyrazole **16** provided a mixture of two regioisomeric pyrazolo[1,5-*a*]pyrimidines.

**Scheme 38:**
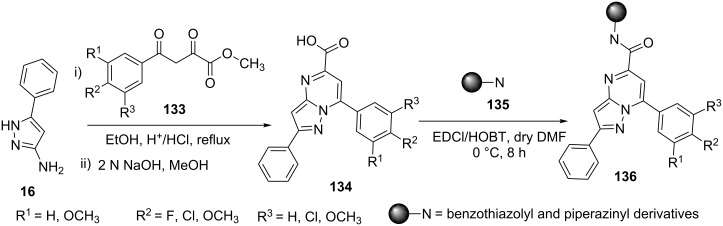
Synthesis of aminobenzothiazole/piperazine linked pyrazolo[1,5-*a*]pyrimidines.

Kamal et al. [94] reported the synthesis of aminobenzothiazole linked pyrazolo[1,5-*a*]pyrimidine conjugates (benzothiazolyl derivatives, **136**). Methyl-2,7-diphenylpyrazolo[1,5-*a*]pyrimidine-5-carboxylates, obtained by the reactions of 5-aminopyrazole **16** with aryl-β-diketoesters **133** in ethanol, were hydrolyzed in presence of methanolic sodium hydroxide to give corresponding carboxylic acids **134**. Aminobenzothiazoles **135** were linked with carboxylic acids **134** to provide the amide derivatives (benzothiazolyl derivatives, **136**) using amide coupling reagent 1-ethyl-3-(3-dimethylaminopropyl)carbodiimide–hydroxybenzotriazole (Scheme 38). The compounds were found to possess good anticancer activity against human colon, leukemia, melanoma cancer cell lines. In another report, Kamal et al. [95] synthesized similar pyrazolo[1,5-*a*]pyrimidinyl amide derivatives (piperazinyl derivatives, **136**) by condensing piperazin-1-yl(pyridin-3-yl)methanone (piperazinyl derivatives, **135**) with **134** which were evaluated for their cytotoxic potential against MCF-7, HeLa, IMR 32 and SiHa cancer cell lines. Pyrazolo[1,5-*a*]pyrimidinyl amide derivatives **136** having piperazinyl derivatives, R^1^ = H, R^2^ = F and OCH_3_, R^3^ = H and OCH_3_, respectively, were found to be the most active compounds showing a minimum survival of the cancer cells.

**Scheme 39:**
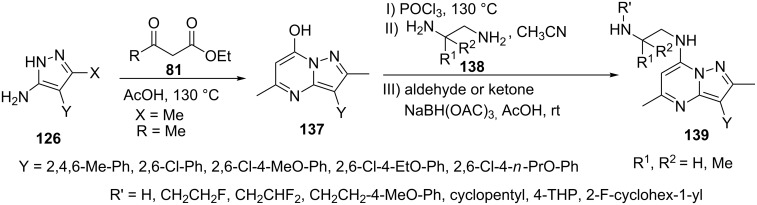
Synthesis of aminoalkylpyrazolo[1,5-*a*]pyrimidine-7-amines.

Griffith et al. [96] described the synthesis of 7-hydroxypyrazolo[1,5-*a*]pyrimidine derivatives **137** by cyclocondensation of 5-aminopyrazole **126** (X = Me, Y = Ar) with ethyl acetoacetate **81**. Pyrazolo[1,5-*a*]pyrimidine derivatives **137** thus obtained were treated with POCl_3_ to give the 7-chloropyrazolo[1,5-*a*]pyrimidine derivative which on coupling with the appropriately substituted diamine derivatives provided aminoalkylpyrazolo[1,5-*a*]pyrimidine-7-amines **139**. Substituted ethylenediamines resulted in the product formed by addition from the less sterically hindered amino group. The free amino group was alkylated by reductive amination on reaction with substituted aldehyde or ketones to provide the corresponding pyrazolo[1,5-*a*]pyrimidine derivatives **139** (Scheme 39). The pyrazolo[1,5-*a*]pyrimidine derivatives **139** were evaluated as neuropeptide NPY Y1R antagonists with high binding affinity and selectivity.

**Scheme 40:**
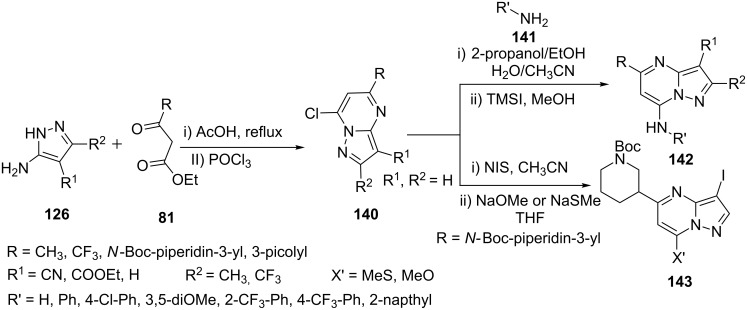
Synthesis of pyrazolo[1,5-*a*]pyrimidines from condensation of 5-aminopyrazole **126** and ethyl acetoacetate.

**Scheme 41:**
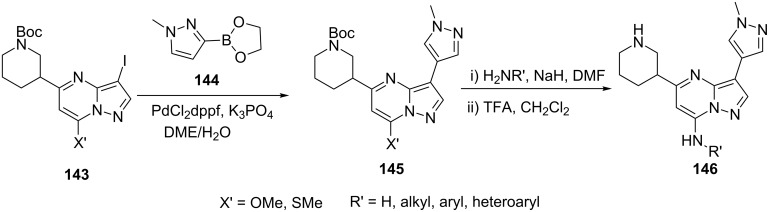
Synthesis of 7-aminopyrazolo[1,5-*a*]pyrimidines.

Using a similar approach Dwyer et al. [97] reported the synthesis of various pyrazolo[1,5-*a*]pyrimidinyl derivatives **142**, **143**, **145** and **146** following a sequence of reactions as depicted in Scheme 40 and Scheme 41. 7-Chloropyrazolo[1,5-*a*]pyrimidines **140** obtained by 4-H/cyano/carboxylate-5-aminopyrazoles **126** (X = H, Y = R) and β-ketoesters **81** followed by chlorination with POCl_3_, were converted to 7-aminopyrazolo[1,5-*a*]pyrimidines **142** and 7-methoxy/thiomethoxypyrazolo[1,5-*a*]pyrimidines **143** on treatment of NIS, NH_3_ **141** in propanol and NaOMe/NaSMe in THF, respectively. Further, compound **143** was coupled with 3-pyrazolylboronate to give 3-pyrazolylpyrazolo[1,5-*a*]pyrimidines **145** and subsequently converted to 7-amino-3-pyrazolylpyrazolo[1,5-*a*]pyrimidines **146** (Scheme 41). The synthesized pyrazolo[1,5-*a*]pyrimidine derivatives were evaluated for their CHK1 kinase inhibitory activity. Pyrazolo[1,5-*a*]pyrimidine derivative **142** with R^1^ = 3-(1-methylpyrazolyl), R^2^ = H, R = 3-pyridyl and R´ = 5-(3-methylthiazolyl) was found to be the most potent, selective CHK1 inhibitor.

Azeredo et al. [98] reported a similar synthesis of 7-arylaminopyrazolo[1,5-*a*]pyrimidines **143** by the coupling reaction of 7-chloropyrazolo[1,5-*a*]pyrimidines **140** with various aryl amines **141** in ethanol, which were evaluated for their anti-Plasmodium falciparum, antimalarial, and Pf-dihydroorotate dehydrogenase inhibitory activity (Scheme 40). 7-Arylaminopyrazolo[1,5-*a*]pyrimidine derivative **142** with R = CF_3_, R^1^ = H, R^2^ = CH_3_ and R´ = 7-β-naphthyl was found to be having highest selectivity and inhibition with IC_50_ = 0.16 ± 0.01 mM.

Synthesis of 7-aminopyrazolo[1,5-*a*]pyrimidines **146** was also reported by Hylsov et al. [99] using an almost similar synthetic procedure by coupling 7-chloropyrazolo[1,5-*a*]pyrimidines **140** with 3-picolylamine in acetonitrile at reflux temperature (Scheme 40).

**Scheme 42:**
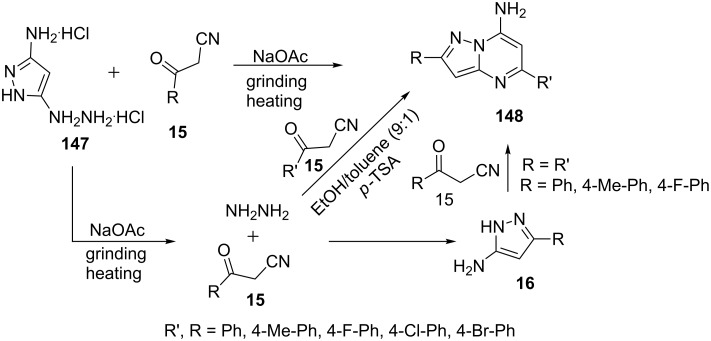
Unexpected synthesis of 7-aminopyrazolo[1,5-*a*]pyrimidines under solvent free and solvent-mediated conditions.

Recently, Aggarwal et al. [100] reported an unexpected synthesis of 7-aminopyrazolo[1,5-*a*]pyrimidine (R’ = R, **148**) from the reaction of 3(5)-amino-5(3)-hydrazinopyrazole dihydrochloride (**147**) with 3-oxo-3-arylpropanenitrile **15** under solvent-free grinding conditions. The reaction was proposed to proceed via formation of hydrazine by C–N bond cleavage which under reaction conditions provided 7-aminopyrazolo[1,5-*a*]pyrimidines **148** on coupling with 3-oxo-3-arylpropanenitrile **15** (Scheme 42). The structure of compounds **148** was established by the combined use of NMR and DFT calculations.

In another report Aggarwal et al. [101] synthesized similar 7-aminopyrazolo[1,5-*a*]pyrimidine **148** from the reaction of hydrazine hydrate with two different 3-oxo-3-arylpropanenitriles **15** which are successively added one after the other in toluene/ethanol (9:1) at reflux temperature in presence of *p*-TSA. The reaction carried out in pure ethanol provided a mixture of 5-aminopyrazoles (Scheme 42). The synthesized 7-aminopyrazolo[1,5-*a*]pyrimidines **148** were found to be good anti-inflammatory agents.

**Scheme 43:**
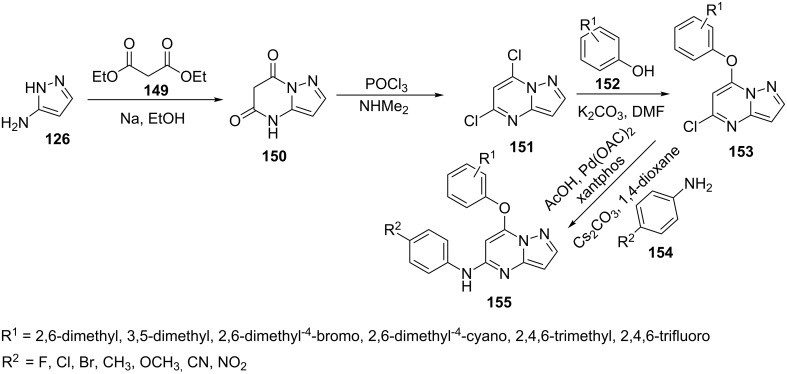
Synthesis of *N*-(4-aminophenyl)-7-aryloxypyrazolo[1,5-*a*]pyrimidin-5-amines.

Tian et al. [102] reported a protocol for the efficient synthesis of pyrazolo[1,5-*a*]pyrimidine-5,7-dione (**150**) by the reaction of 5-aminopyrazole (**126**) with diethyl malonate (**149**). Pyrazolo[1,5-*a*]pyrimidine-5,7-dione (**150**) was chlorinated to give 5,7-dichloropyrazolo[1,5-*a*]pyrimidine (**151**) which subsequently coupled with various phenols **152** at the more reactive 7-position under mild reaction conditions in presence of K_2_CO_3_ in acetic acid/DMF to give pyrazolo[1,5-*a*]pyrimidine derivative **153**. Various aromatic amines **154** were then coupled at 5-postion under Buchwald–Hartwig conditions to get the desired 5-aminopyrazolo[1,5-*a*]pyrimidine derivatives **155** (Scheme 43). All the synthesized compounds were screened for their anti-HIV activities in MT4 cell cultures and compound **155** (R^1^ = 2,4,6-trimethyl and R^2^ = 4-cyano) was found as most inhibiting for HIV-1 replication having an EC_50_ = 0.070 μM and the SI (selectivity index) = 3999, which were better than the drugs NVP (nevirapine) (EC_50_ = 0.17 μM) and DLV (delavirdine) (EC_50_ = 0.16 μM).

**Scheme 44:**
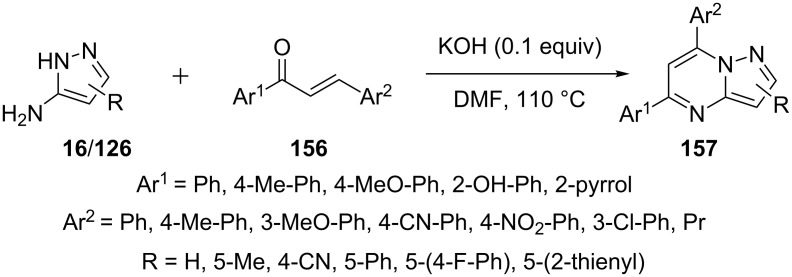
Base-catalyzed synthesis of 5,7-diarylpyrazolo[1,5-*a*]pyrimidines.

Kaswan et al. [103] reported the reaction of 5-aminopyrazoles **16/126** with chalcones **156** in DMF in the presence of inorganic base KOH for the synthesis of 5,7-diarylpyrazolo[1,5-*a*]pyrimidines **157**. Chalcones **156** with electron-withdrawing group like nitro, cyano on *para-*position of aryl or heteroaryl ring and 2-hydroxyphenyl group resulted in lower yields as compared to chalcones with electron-donating groups (Scheme 44).

**Scheme 45:**
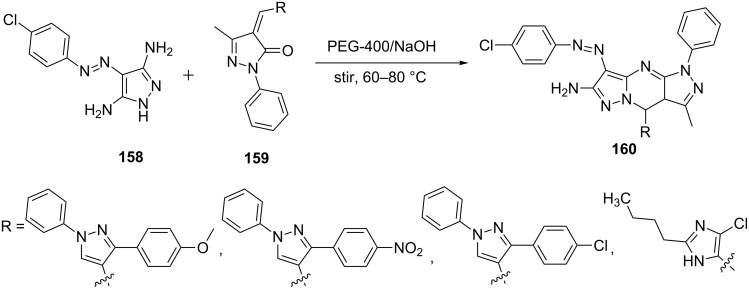
Synthesis of 6,7-dihydropyrazolo[1,5-*a*]pyrimidines in PEG-400.

Chobe et al. [104] reported the reaction of 3,5-diamino-4-diazopyrazole derivative **158** with 4-substituted benzylidene-3-methyl-1*H*-pyrazol-5(4*H*)-one **159** in PEG-400. The reaction resulted in the synthesis of 6,7-dihydropyrazolo[1,5-*a*]pyrimidine derivatives **160** (Scheme 45). Selected compounds were studied for their interaction with calf thymus DNA using various techniques like electronic spectra, viscosity measurement and thermal denaturation.

**Scheme 46:**
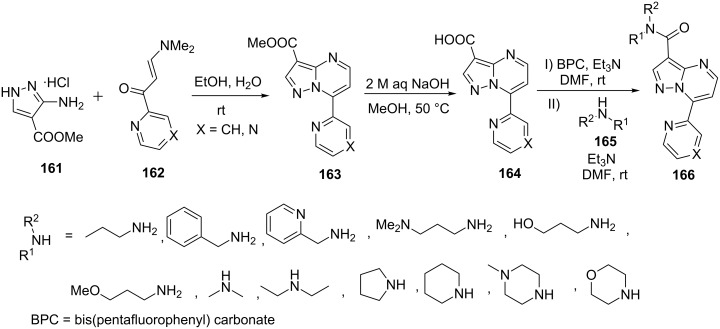
Synthesis of 7-heteroarylpyrazolo[1,5-*a*]pyrimidine-3-carboxamides.

Ahmetaj et al. [105] described a simple and efficient protocol for the synthesis of 7-heteroarylpyrazolo[1,5-*a*]pyrimidine-3-carboxamides **166** from the reaction of 5-aminopyrazole **161** with (*E*)-3-(dimethylamino)-1-(heteroaryl)prop-2-en-1-one **162** in aqueous ethanol at ambient temperature through the intermediacy of methyl 7-heteroarylpyrazolo[1,5-*a*]pyrimidine-3-carboxylates **163** which was subsequently hydrolyzed to give the corresponding carboxylic acids **164** followed by coupling with various primary and secondary amines **165** in presence of bis(pentafluorophenyl) carbonate (BPC) as activating agent (Scheme 46).

**Scheme 47:**
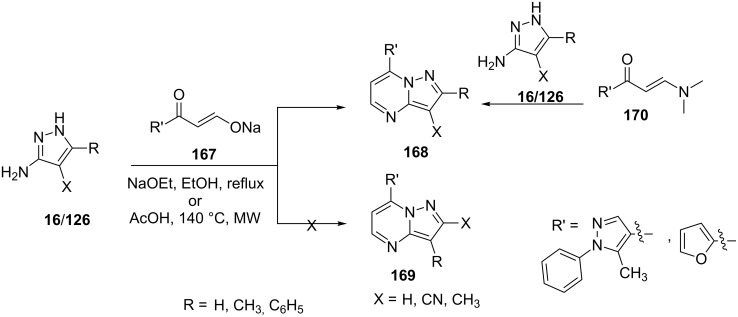
Synthesis of 7-heteroarylpyrazolo[1,5-*a*]pyrimidine derivatives under conventional heating and microwave assistance.

Abdelhamid et al. [106] reported the synthesis of pyrazolo[1,5-*a*]pyrimidine derivatives **168** from the reaction of 5-aminopyrazoles **16/126** with sodium 3-(5-methyl-1-phenyl-1*H*-pyrazol-4-yl)-3-oxoprop-1-en-1-olate (**167**) with high regioselectivity without any traces of other possible regioisomeric pyrazolo[1,5-*a*]pyrimidines **169**. The regioselectivity of the reaction was attributed to the higher nucleophilicity of the exocyclic primary amino group over the endocyclic amino group. Synthesis of pyrazolo[1,5-*a*]pyrimidine derivatives **168** was also achieved by an alternate route with equal ease by the reaction of 5-aminopyrazoles **16/126** with 3-(dimethylamino)-1-(5-methyl-1-phenyl-1*H*-pyrazol-4-yl)prop-2-en-1-ones (**170**) for structural confirmations (Scheme 47).

Recently, Abdelhamid et al. [107] also reported the synthesis of 7-(furan-2-yl)-2-phenylpyrazolo[1,5-*a*]pyrimidine (**168**) using a similar synthetic strategy from the reaction of 5-aminopyrazole **16/126** with sodium 3-(furan-2-yl)-3-oxoprop-1-en-1-olate (**167,** Scheme 47).

**Scheme 48:**
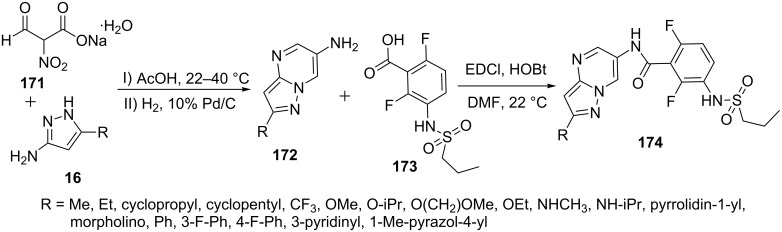
Synthesis of *N*-aroylpyrazolo[1,5-*a*]pyrimidine-5-amines.

Ren et al. [108] described the synthesis of 6-aminopyrazolo[1,5-*a*]pyrimidine derivatives **172** involving the condensation of 5-aminopyrazole derivative **16** and sodium nitromalonaldehyde **171** followed by reduction of the nitro group by hydrogenation to give 6-aminopyrazolo[1,5-*a*]pyrimidines **172**. 6-Aminopyrazolo[1,5-*a*]pyrimidines **172** thus obtained were coupled with variously substituted benzoic acids **173** to give corresponding amide derivatives of pyrazolo[1,5-*a*]pyrimidines **174** (Scheme 48). Some of the compounds were found to be potent, selective and orally available B-Raf inhibitors with favorable physicochemical and pharmacokinetic properties.

**Scheme 49:**
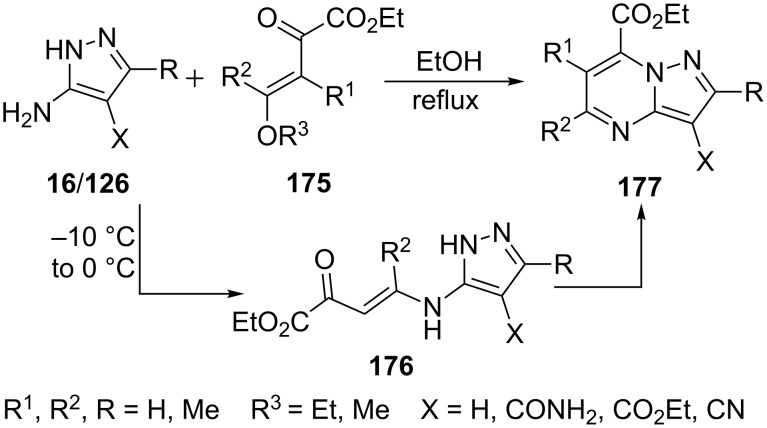
Regioselective synthesis of ethyl pyrazolo[1,5-*a*]pyrimidine-7-carboxylate.

Stepaniuk et al. [109] reported the reaction of 5-aminopyrazole derivatives **16/126** with β,γ-unsaturated-γ-alkoxy-α-ketoesters **175** for the regioselective synthesis of pyrazolo[1,5-*a*]pyrimidines **177** in refluxing ethanol. The reaction provided high regioselectivity compared to other 1,3-dielectrophiles like 1,3-dicarbonyl compounds. The reaction was proposed to proceed through intermediate **176** which was isolated at −10 °C to 0 °C but was found to be unstable even at room temperature (Scheme 49).

**Scheme 50:**
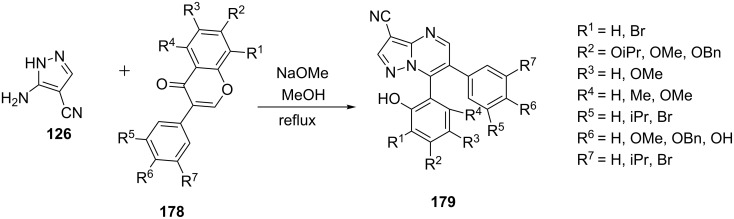
Sodium methoxide-catalyzed synthesis of 3-cyano-6,7-diarylpyrazolo[1,5-*a*]pyrimidines.

Ma et al. [110] reported the synthesis of 3-cyano-6,7-diarylpyrazolo[1,5-*a*]pyrimidines **179** comprising the reaction of 1.5 equivalents of 5-aminopyrazole **126** with 1 equivalent of isoflavones **178** in the presence of 3 equivalents of sodium methoxide in methanol (Scheme 50). The method has the merits of being simple in operation with mild reaction conditions and good yields of fused pyrazole derivatives.

### Synthesis of pyrazolo[3,4-*d*]pyrimidines

A diversity of biological effects is associated with pyrazolo[3,4-*d*]pyrimidines. They are known to exhibit antiviral [111–112], pesticidal [113], anti-inflammatory [114], antimicrobial [115–117], antileukemic [118], antitumor [114,119–120], pan-RAF inhibiting [121], tyrosine kinase RET inhibiting [122], CNS [123], cardiovascular [124–125] and tuberculostatic [126–127] activities. The promising therapeutic potential of pyrazolo[3,4-d]pyrimidines prompted researchers to develop novel synthetic strategies to provide this class of compounds.

**Scheme 51:**
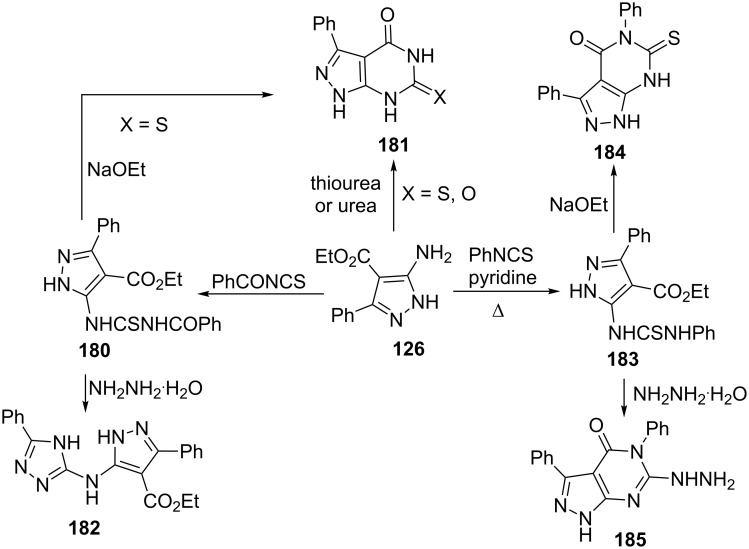
Synthesis of various pyrazolo[3,4-*d*]pyrimidine derivatives.

Ghozlan et al. [128] reported the reactions of ethyl 5-amino-3-phenylpyrazole-4-carboxylate (**126**) with benzoylisothiocyanate or phenylisothiocyantes for the synthesis of *N*-thiocarbamoyl pyrazole derivatives **180** and **183** which gave pyrazolo[3,4-*d*]pyrimidine derivatives **181** and **184** on treatment with ethanolic sodium ethoxide. Pyrazolo[3,4-*d*]pyrimidine derivatives **181** were also obtained directly by fusion of thiourea/urea with 5-aminopyrazole **126** in an oil bath at 120 °C. *N*-Thiocarbamoyl pyrazole derivatives **180** and **183** underwent cyclization with hydrazine hydrate to give 5-(*N*-triazolyl)aminopyrazole derivative **182** and hydrazinopyrazolo[3,4-*d*]pyrimidines **185**, respectively (Scheme 51).

**Scheme 52:**
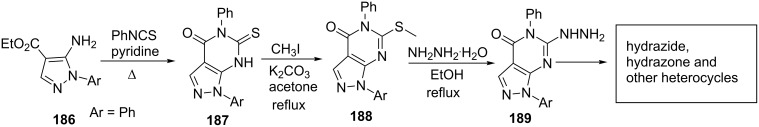
Synthesis of hydrazinopyrazolo[3,4-*d*]pyrimidine derivatives.

El-Moghazy et al. [129] described the synthesis of pyrazolo[3,4-*d*]pyrimidine derivatives **187** using a similar approach by the reaction of ethyl 5-amino-1-phenyl-1*H*-pyrazolo-4-carboxylate (**186**) and phenyl isothiocyanate in pyridine. The pyrazolo[3,4-*d*]pyrimidine derivative **187** thus obtained was methylated with iodomethane in acetone in the presence of potassium carbonate to give methyl thioether **188** which provided hydrazinopyrazolo[3,4-*d*]pyrimidines **189** on treatment with an excess of hydrazine hydrate in ethanol. The hydrazine derivative **189** thus obtained was made to react with several carbonyl compounds like aldehydes, benzoyl chloride and ethyl acetoacetate to append hydrazone, carbohydrazide and pyrazolone type moieties on pyrazolo[3,4-*d*]pyrimidine. Further, hydrazinyl derivative **189** provided various fused triazolylpyrazolo[3,4-*d*]pyrimidines on treatment with various reagents like aliphatic acids, benzoyl chlorides, chloroacetyl chloride, isothiocyanate and carbon disulfide under appropriate reaction conditions (Scheme 52).

**Scheme 53:**
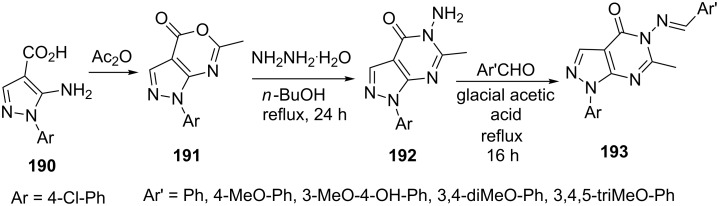
Synthesis of *N*-arylidinepyrazolo[3,4-*d*]pyrimidin-5-amines.

Hassan et al. [130] reported the synthesis of various pyrazolo[3,4-*d*]pyrimidine derivatives **193** (Scheme 53). 5-Aminopyrazole-4-carboxylic acid **190** on refluxing in acetic anhydride provided pyrazolooxazinones **191** which converted to 5-aminopyrazolo[3,4-*d*]pyrimidine **192** by reaction with hydrazine hydrate in *n*-butanol. Further treatment of **192** with aromatic aldehydes provided the corresponding Schiff base **193**.

**Scheme 54:**
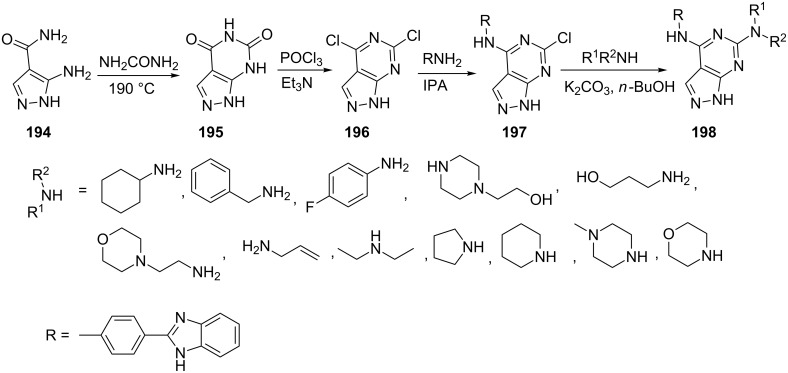
Synthesis of pyrazolo[3,4-*d*]pyrimidinyl-4-amines.

Singla et al. [131] reported the synthesis of pyrazolo[3,4-*d*]pyrimidinyl-4-amines **198** possessing 4-(1*H*-benzimidazol-2-yl)phenylamine moiety at C4 position and primary as well as secondary amines at C6 position starting from 5-aminopyrazole-4-carboxamide (**194**). Compound **194** was treated with urea to give 1*H*-pyrazolo[3,4-*d*]pyrimidine-4,6(5*H*,7*H*)-dione (**195**) followed by chlorination with POCl_3_ to furnish 4,6-dichloropyrazolo[3,4-*d*]pyrimidine (**196**) which on coupling with the corresponding amines provided [4-(1*H*-benzimidazol-2-yl)-phenyl]-(6-substituted-1*H*-pyrazolo[3,4-*d*]pyrimidin-4-yl)-amines **198** (Scheme 54). Pyrazolo[3,4-*d*]pyrimidine derivatives **198** were evaluated for their antitumor activities against various human cancer cell lines. The compound **198** with a pyrrolidine moiety was identified as most potent and promising member as it showed superior antitumor activities over other derivatives.

**Scheme 55:**
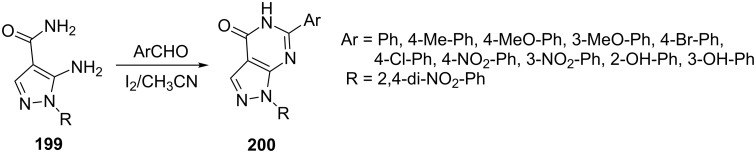
Iodine-catalyzed synthesis of pyrazolo[3,4-*d*]pyrimidinones.

Bakavoli et al. [115] reported the synthesis of pyrazolo[3,4-*d*]pyrimidine derivatives **200** from the cyclocondensation of 5-amino-1-(2,4-dinitrophenyl)-1*H*-pyrazole-4-carboxamide (**199**) with aromatic aldehydes in the presence of iodine in acetonitrile (Scheme 55). The synthesized pyrazolo[3,4-*d*]pyrimidines were evaluated for antibacterial activities.

**Scheme 56:**
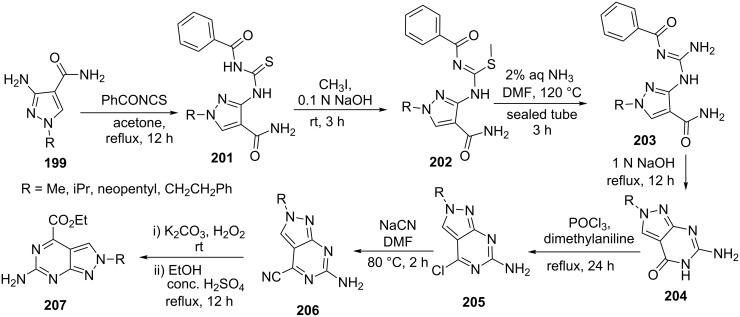
Synthesis of ethyl 6-amino-2*H*-pyrazolo[3,4-*d*]pyrimidine-4-carboxylate.

Venkatesan et al. [132] also used 4-carboxamide-5-aminopyrazole **199** for the synthesis of pyrazolo[3,4-*d*]pyrimidines **207** (Scheme 56). The condensation of **199** with benzoyl isothiocyanate under reflux conditions in dry acetone provided benzoylthioureido derivatives **201** which were converted to methylthio derivative **202** with iodomethane in aqueous sodium hydroxide solution at ambient temperature. The methylthio group was converted to benzoylguanidino derivative **203** by nucleophilic displacement with ammonia in DMF on vigorous heating in a sealed tube. Subsequently, compounds **203** were converted to 6-amino-2-substituted-2*H*-pyrazolo[3,4-*d*]pyrimidin-4(5*H*)-one derivatives **204** by refluxing in 1 N sodium hydroxide. Pyrazolo[3,4-*d*]pyrimidinone **204** were further chlorinated by phosphorus oxychloride and subsequently converted to carboxylic esters **207** via cyanation followed by hydrolysis and esterification.

**Scheme 57:**
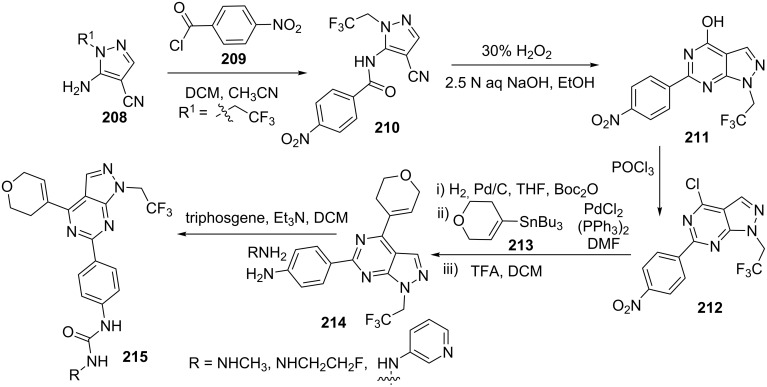
Synthesis of 4-substituted-(3,6-dihydropyran-4-yl)-1*H*-pyrazolo[3,4-*d*]pyrimidines.

Kaplan et al. [20] explored the synthesis of pyrazolo[3,4-*d*]pyrimidine derivatives starting from 5-amino-4-cyanopyrazoles **208** (Scheme 57). 5-Amino-4-cyanopyrazole **208** was benzoylated with *p*-nitrobenzoyl chloride (**209**) and subsequently cyclized to pyrazolo[3,4-*d*]pyrimidine derivative **211** by refluxing in sodium hydroxide and hydrogen peroxide. Chlorination of pyrazolo[3,4-*d*]pyrimidine derivative **211** with phosphorus oxychloride afforded 4-chloro-1*H*-pyrazolo[3,4-*d*]pyrimidine derivative **212**. The chloro and nitro groups were manipulated to introduce a 3,6-dihydropyran group at position-4 by Stille reaction which provided 4-(4-(3,6-dihydropyran-4-yl)-1*H*-pyrazolo[3,4-*d*]pyrimidin-6-yl)aniline **214** by NO_2_ group reduction with H_2_, Pd/C followed by Boc protection, coupling with tributyl(3,6-dihydro-2*H*-pyran-4-yl)stannane (**213**) and subsequent Boc deprotection with TFA in DCM. Pyrazolo[3,4-*d*]pyrimidinylaniline **214** was used to synthesize pyrazolo[3,4-*d*]pyrimidinylureas **215** on treatment with triphosgene and corresponding amines.

**Scheme 58:**
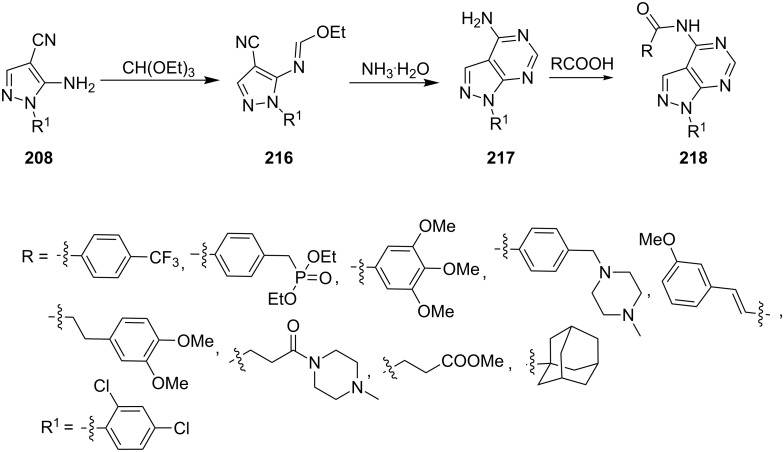
Synthesis of 1-(2,4-dichlorophenyl)pyrazolo[3,4-*d*]pyrimidin-4-yl carboxamides.

Liu et al. [133] reported the synthesis of 4-amino-1-(2,4-dichlorophenyl)pyrazolo[3,4-*d*]pyrimidine derivatives **217** by the reaction of ethyl *N*-(4-cyano-1-(2,4-dichlorophenyl)-1*H*-pyrazol-5-yl)formimidate (**216**) with ammonia. *N*-(4-Cyano-1-(2,4-dichlorophenyl)-1*H*-pyrazol-5-yl)formimidate (**216**), in turn was obtained by reaction of 5-amino-1-(2,4-dichlorophenyl)-1*H*-pyrazole-4-carbonitrile (**208**) with trimethylorthoformate. 4-Amino-1-(2,4-dichlorophenyl)pyrazolo[3,4-*d*]pyrimidine derivatives **217** were coupled with various carboxylic acids in the presence of EDCl, DMAP and HOBt in *N*,*N*-dimethylformamide at room temperature for the synthesis of the corresponding amide derivatives **218** (Scheme 58).

**Scheme 59:**
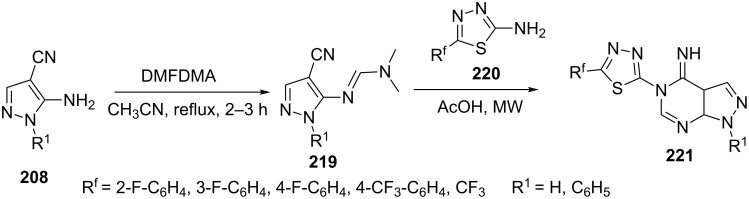
Synthesis of 5-(1,3,4-thidiazol-2-yl)pyrazolo[3,4-*d*]pyrimidine.

Song et al. [134] explored the synthesis of pyrazolo[3,4-*d*]pyrimidine derivatives **221** through the intermediacy of amidines **219** obtained by reaction of 5-amino-4-cyanopyrazole **208** with *N*,*N*-dimethylformamide dimethyl acetal (DMFDMA) in acetonitrile at reflux temperature. Amidines **219** were condensed with appropriate 2-amino-5-subsitituted-1,3,4-thiadiazoles **220** under microwave irradiation in acetic acid for the generation of the desired pyrazolo[3,4-*d*]pyrimidine derivatives **221** (Scheme 59). The synthesized pyrazolo[3,4-*d*]pyrimidines **221** were proved to be good anticancer agents by MTT assay against HL-60 cancer cell lines.

**Scheme 60:**
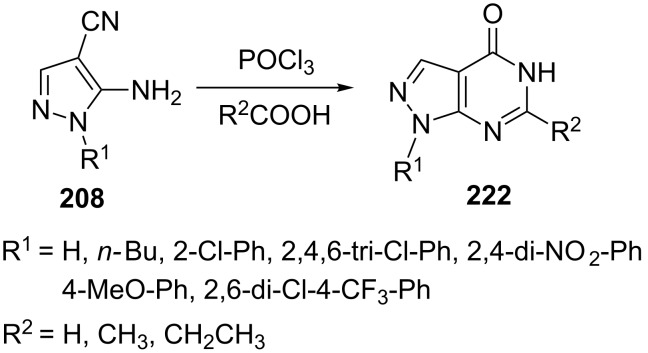
One pot POCl_3_-catalyzed synthesis of 1-arylpyrazolo[3,4-*d*]pyrimidin-4-ones.

Zhang et al. [135] reported the reaction of 5-amino-4-cyanopyrazole **208** and aliphatic acids (R^2^COOH) in the presence of POCl_3_ to afford the respective 1-arylpyrazolo[3,4-*d*]pyrimidin-4-ones **222** in a one pot single step procedure (Scheme 60). POCl_3_ acted as chlorinating agent as well as an oxidant in the reaction which in situ generated acyl chlorides from acids making the condensation and cyclization easier and faster.

**Scheme 61:**
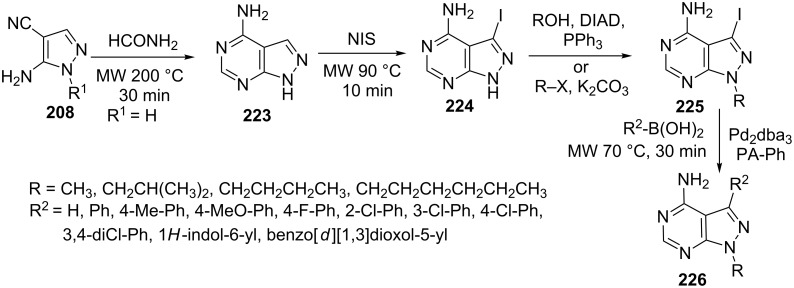
Synthesis of 4-amino-N1,C3-dialkylpyrazolo[3,4-*d*]pyrimidines under Suzuki conditions.

The reaction of 5-amino-4-cyanopyrazole (**208**) and formamide was carried out by Todorovic et al. [136] under microwave irradiation at 200 °C to give 4-aminopyrazolo[3,4-*d*]pyrimidine (**223**) which on iodination with *N*-iodosuccinimide followed by *N*1-alkylation (Mitsunobu or substitution) provided corresponding *N*1-alkyl-3-iodopyrazolo[3,4-*d*]pyrimidine derivatives **225**. The iodinated pyrazolo[3,4-*d*]pyrimidines were alkylated at C3 with boronic acids (R_2_-B(OH)_2_) using Suzuki coupling conditions to give 4-amino-*N*1,*C*3-dialkylpyrazolo[3,4-*d*]pyrimidines **226** (Scheme 61).

### Synthesis of pyrazolo[3,4-*b*]pyrazines

Pyrazolo[3,4-*b*]pyrazines have received great attention because of their interesting biological activities such as inhibition of protein kinases [137], blood platelet aggregation [138], bone metabolism improvers [139] as well as antifungal [140], antibacterial [141], antiparasitic [142] and antiviral [143] activity. There are several methods reported in literature for the construction of pyrazolo[3,4-*b*]pyrazine nucleus.

**Scheme 62:**
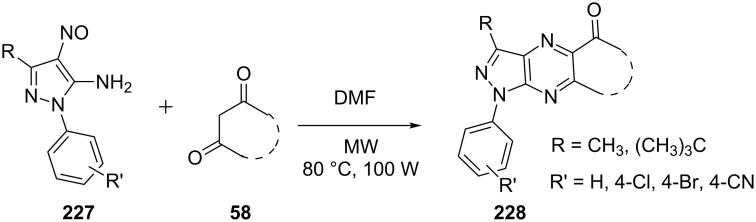
Microwave-assisted synthesis of pyrazolo[3,4-*b*]pyrazines.

Quiroga et al. [144] studied the reaction of *o*-aminonitrosopyrazoles **227** and cyclic β-diketones **58** in various solvents like pyridine, acetic acid and *N*,*N*-dimethylformamide for the synthesis of pyrazolo[3,4-*b*]pyrazines **228** (Scheme 62). No measurable product was observed in acetic acid and pyridine but reaction in DMF provided promising results with good yields of the pyrazolo[3,4-*b*]pyrazines **228** in short reaction time. The reaction under microwave irradiation (100 W at 80 °C) in DMF provided the desired product in 85% yield in just 9 min. Easy work-up, mild reaction conditions and good yields makes this protocol a simple procedure for the synthesis of pyrazolo[3,4-*b*]pyrazines.

**Scheme 63:**
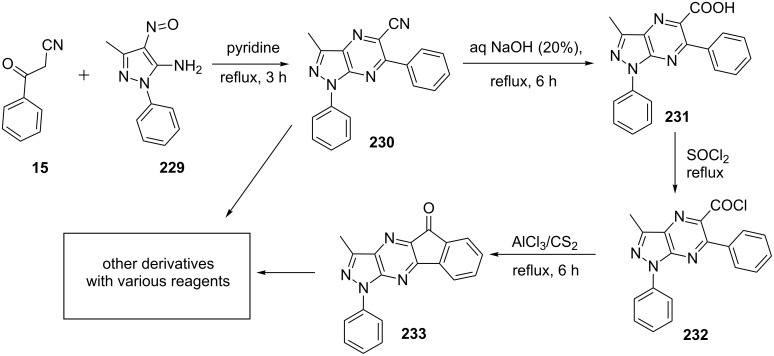
Synthesis and derivatization of pyrazolo[3,4-*b*]pyrazine-5-carbonitriles.

Emary et al. [145] described the cyclocondensation of 5-amino-4-nitrosopyrazoles **229** and β-ketonitriles **15** in pyridine to give 1,3,6-trisubstitutedpyrazolo[3,4-*b*]pyrazine-5-carbonitriles **230** in good yields. 5-Carbonitrilepyrazolo[3,4-*b*]pyrazines **230** were hydrolyzed to corresponding pyrazolo[3,4-*b*]pyrazine-5-carboxylic acids **231** and subsequently converted to acid chloride **232** at reflux temperature with thionyl chloride (SOCl_2_) which underwent intramolecular Friedel–Crafts reaction in presence of Lewis acid to give 3-methyl-1-phenyl-1*H*-indeno[2,1-*e*]pyrazolo[3,4-*b*]pyrazin-5-one (**233**). Compound **233** was used to synthesize several other indenopyrazolopyrazinone derivatives by reaction with active methylene compounds, aromatic amines, hydroxylamine hydrochloride, semicarbazide hydrochloride, thiosemicarbazide, hydrazine hydrate and phenyl hydrazine (Scheme 63).

Similarly, Farghley et al. [146] reported the reaction of 5-amino-4-nitrosopyrazoles **229** and β-ketonitriles **15** in pyridine for the synthesis of pyrazolo[3,4-*b*]pyrazines **230** which was used as synthetic precursor to generate several other substituted pyrazolo[3,4-*b*]pyrazine derivatives via amidoxime and carbohydrazide intermediates obtained from the reaction of appropriate substrates with nitrile group (Scheme 63).

### Synthesis of pyrazolo[1,5-*a*][1,3,5]triazine

Pyrazolo[1,5-*a*][1,3,5]triazine is a well-known class of fused pyrazole derivatives with a broad spectrum of biological activities such as anticancer [147], anti-inflammatory [148], anxiolytic [149], anticonvulsant [150] and antidepressant [151]. Accordingly, a large number of synthetic methods have been reported for the construction of pyrazolo[1,5-*a*][1,3,5]triazine derivatives, out of which condensation of the 5-aminopyrazoles with ethoxycarbonyl isothiocyanate/ethoxycarbonyl isocyanates is the most common method for their synthesis.

**Scheme 64:**
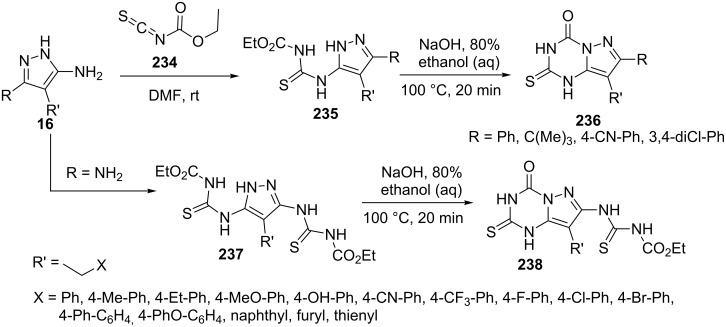
Synthesis of 2-thioxo-pyrazolo[1,5-*a*][1,3,5]triazin-4-ones.

Bera et al. [152] reported the synthesis of 2-thioxo-pyrazolo[1,5-*a*][1,3,5]triazin-4-ones **236** and **238** via annulation of 1,3,5-triazine ring onto 5-aminopyrazoles. The reactions of 5-aminopyrazoles **16** with ethoxycarbonyl isothiocyanate **234** was carried out in DMF to give thiourea derivatives **235** which on treatment with NaOH in ethanol underwent cyclization to give 2-thioxo-pyrazolo[1,5-*a*][1,3,5]triazin-4-ones **236** (Scheme 64). 3,5-Diaminopyrazoles (R = NH_2_, **15**) following the same reaction sequence led to the formation of 2-thioxopyrazolo[1,5-*a*][1,3,5]triazin-4-one-6-thiourea derivative **238** through the intermediacy of bithiourea **237** (Scheme 64).

**Scheme 65:**
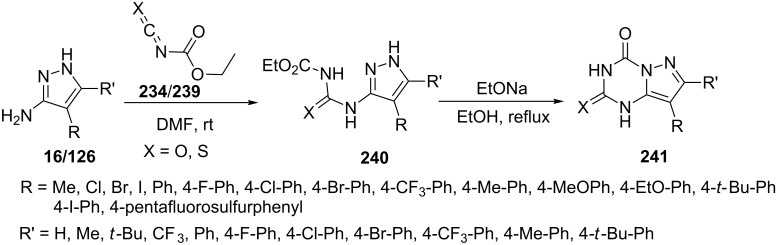
Synthesis of 2,3-dihydropyrazolo[1,5-*a*][1,3,5]triazin-4(1*H*)-one.

Sun et al. [24] reported the synthesis of 7/8-substituted-2-oxo/thioxo-2,3-dihydropyrazolo[1,5-*a*][1,3,5]triazin-4(1*H*)-one **241** from the reaction of 3/4-substituted-5-aminopyrazoles **16/126** with ethoxycarbonyl isothiocyanate/ethoxycarbonyl isocyanate **234**/**239**, respectively (Scheme 65). This two-step procedure involves the intermediacy of ethoxycarbonyl isocyanate to give *N*-ethoxycarbonyl-*N*’-(pyrazol-3-yl)ureas/thioureas **240** followed by their intramolecular cyclization with sodium ethoxide. The reaction of ethoxycarbonyl isothiocyanate provided higher yields in short time as compared to the reaction of ethoxycarbonyl isocyanate.

**Scheme 66:**
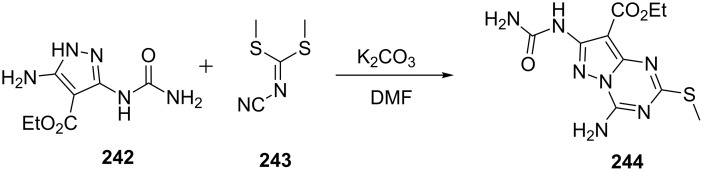
Synthesis of pyrazolo[1,5-*a*][1,3,5]triazine-8-carboxylic acid ethyl ester.

Bakr et al. [153] reported the synthesis of pyrazolo[1,5-*a*][1,3,5]triazine-8-carboxylic acid ethyl ester **244** from the reaction of aminopyrazolylurea derivative **242** and *N*-bis(methylthio)methylenecyanamide (**243**) out in DMF in presence of potassium carbonate (Scheme 66).

**Scheme 67:**
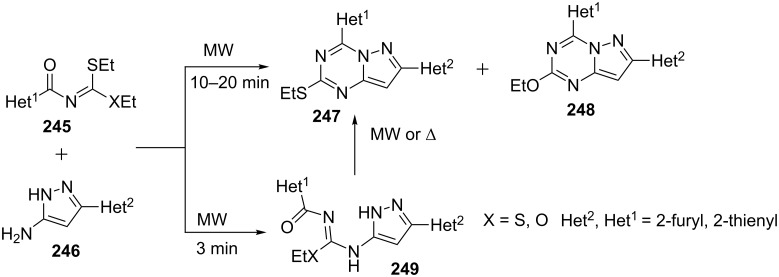
Microwave-assisted synthesis of 4,7-dihetarylpyrazolo[1,5-*a*][1,3,5]triazines.

Insuasty et al. [154] reported the reaction of 5-aminopyrazoles **246** with thiocarbonates (**245**, X = O) and dithiocarbonates (**245**, X = S) under solvent-free conditions using microwave irradiation (300 W, 160–180 °C, 10–20 min) to yield a mixture of two products which were characterized as 2-ethylthio/ethoxy-4,7-dihetarylpyrazolo[1,5-*a*][1,3,5]triazines **247** and **248**. When the reaction was carried out only for 3 min under similar reaction conditions two isomeric intermediates namely ethyl *N*’-(heteroaryl-1-carbonyl)-*N*-(3-heteroaryl-1*H*-pyrazol-5-yl)carbamimidothioate/carbamimidate **249** were isolated successfully in 10% and 24% yields, respectively (Scheme 67). The reactions were also carried under solvent mediated conventional heating conditions which required longer time for completion and provided lower yields of the products.

**Scheme 68:**
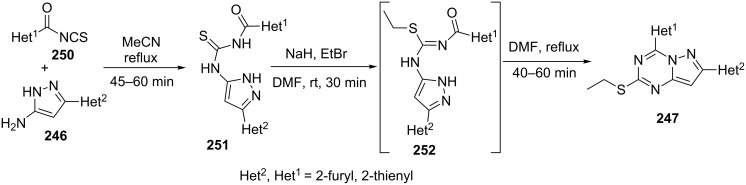
Alternative synthetic route to 4,7-diheteroarylpyrazolo[1,5-*a*][1,3,5]triazines.

In an alternative two step path, thiourea derivatives **251** obtained by reaction of 5-aminopyrazoles **246** with heteroarylisothiocyanates **250** were treated with ethyl bromide in presence of sodium hydride in DMF to generate the intermediate isothioureas **252** which on in situ heating provided target diheteroarylpyrazolo[1,5-*a*][1,3,5]triazines **247** (Scheme 68).

**Scheme 69:**
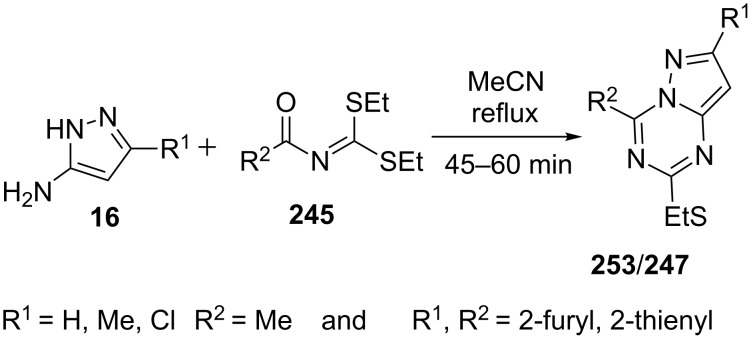
Synthesis of 4-aryl-2-ethylthio-7-methylpyrazolo[1,5-*a*][1,3,5]triazines.

In another report, Insuasty et al. [155] utilized *S*,*S*-diethyl aroyliminodithiocarbonates **245** for condensation with 5-amino-3-methylpyrazole (**16**) to afford 4-aryl-2-ethylthio-7-methylpyrazolo[1,5-a][1,3,5]triazines **247**/**253** (Scheme 69). Synthesized pyrazolo[1,5-*a*][1,3,5]triazines **247**/**253** were evaluated for their anticonvulsant profile by exposing on to electrical and chemical experimental seizures induced in ICR albino mice. Pyrazolo[1,5-*a*][1,3,5]triazines **247** having R^1^,R^2^ = 2-thienyl, showed a good dose-dependent response in the 50, 150 and 300 mg/kg, p.o., range (3/7, 4/7, 5/7; p < 0.05).

**Scheme 70:**
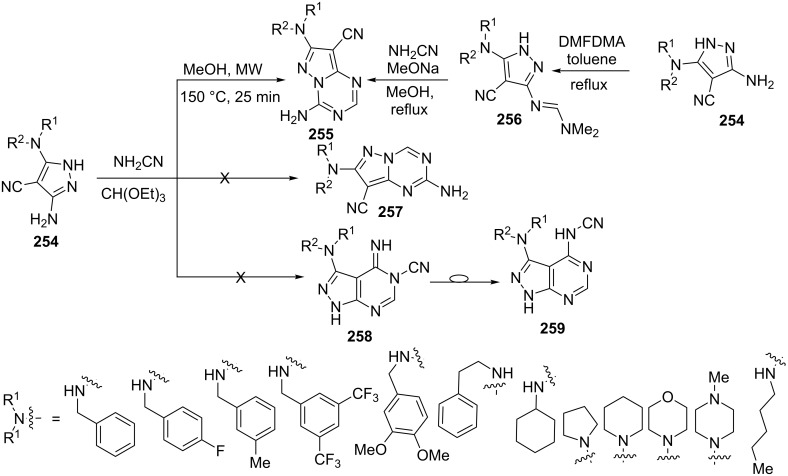
Microwave-assisted synthesis of 4-aminopyrazolo[1,5-*a*][1,3,5]triazine.

Lim et al. [156] studied the reaction of 3,5-diaminopyrazole derivative **254**, cyanamide and triethyl orthoformate in methanol under microwave irradiation (Scheme 70). The reaction resulted in exclusive formation of 4-aminopyrazolo[1,5-*a*][1,3,5]triazine-8-carbonitriles **255** barring the possibilities of isomeric 2-aminopyrazolo[1,5-*a*][1,3,5]triazine **257** and pyrazolo[3,4-*d*]pyrimidine derivatives **258** or **259**. The formation of 4-aminopyrazolo[1,5-*a*][1,3,5]triazine derivative **255** was also confirmed by step-wise annulation of a triazine ring with a predisposed position of the amino group by converting 5-aminopyrazole **254** into the corresponding formamidine **256** on treatment with *N*,*N*-dimethylformamide dimethyl acetal (DMFDMA) and their subsequent condensation with cyanamide in the presence of sodium methoxide. The one-pot multicomponent method provided two times higher yields than the stepwise method.

### Synthesis of pyrazolo[3,4-*d*][1,2,3]triazine

Pyrazolo[3,4-*d*][1,2,3]triazines are important fused pyrazole derivatives because of their biological activity and are valuable synthons in organic transformations. These are also structural analogues of adenosine and guanosine [157–158]. But surprisingly, only a few literature reports are available for synthesis and biological potential of this nucleus.

**Scheme 71:**
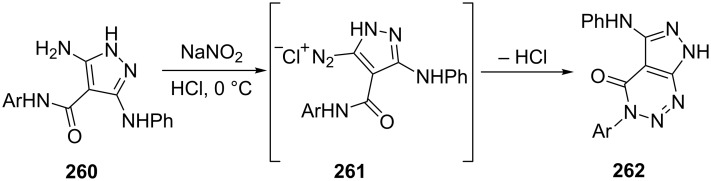
Synthesis of pyrazolo[3,4-*d*][1,2,3]triazines from pyrazol-5-yl diazonium salts.

Pyrazolo[3,4-*d*][1,2,3]triazines **262** were synthesized by Rabie et al. [159] from the diazotization of 4-(*N*-arylcarboxamide)-3-(*N*-phenyl)-3,5-diaminopyrazole derivatives **260** with sodium nitrite which underwent in situ cyclization (Scheme 71).

### Synthesis of pyrazolo[3,4-*e*][1,2,4]triazines

**Scheme 72:**
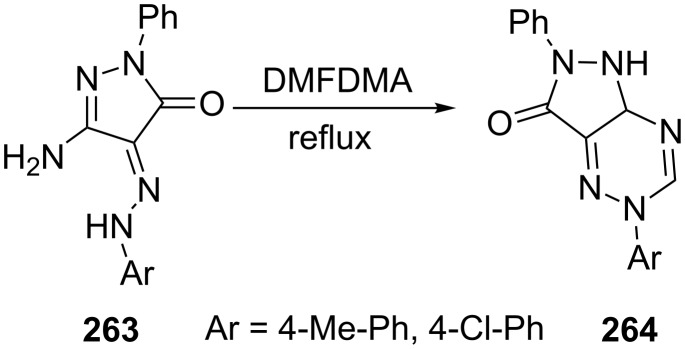
Synthesis of 2,5-dihydropyrazolo[3,4-*e*][1,2,4]triazines.

Matar et al. [160] reported that 3-amino-4-phenylhydrazono-1-phenyl-2-pyrazolin-5-ones **263** undergo cyclization on refluxing in DMFDMA to afford 2,5-dihydropyrazolo[5,1-*c*][1,2,4]triazines **264** in good yields (Scheme 72).

### Synthesis of pyrazolo[5,1-*c*][1,2,4]triazines

The pyrazolo[5,1-*c*][1,2,4]triazine nucleus is present in compounds possessing a variety of pharmacological and biological activities [161–163]. The association of biological properties with this nucleus stimulated the interest of organic chemists in the development of novel synthetic approaches for the construction of the pyrazolo[5,1-*c*][1,2,4]triazine nucleus.

**Scheme 73:**
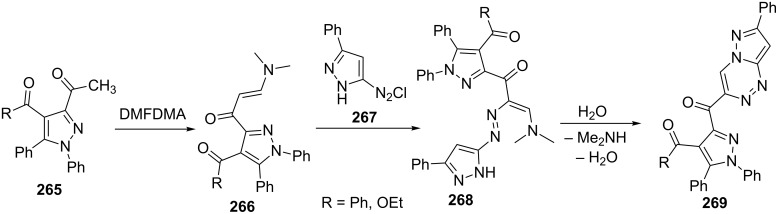
Synthesis of pyrazolo[5,1-*c*][1,2,4]triazines via diazopyrazolylenaminones.

Shawali et al. [164] have reported the synthesis of 3-[(4,5-disubstituted-pyrazol-3-yl)carbonyl]-pyrazolo[5,1-*c*][1,2,4]triazines **269** by cyclization of diazopyrazolylenaminones **268**. 3-Acetylpyrazoles **265** on condensation with DMFDMA provided enaminones **266** which were converted to diazopyrazolylenaminones **268** on coupling with 3-phenylpyrazolyldiazonium salt **267** in pyridine at 0–5 °C. Diazopyrazolylenaminones **268** underwent cyclization under reaction conditions to give pyrazolo[5,1-*c*][1,2,4]triazines **269** (Scheme 73).

**Scheme 74:**
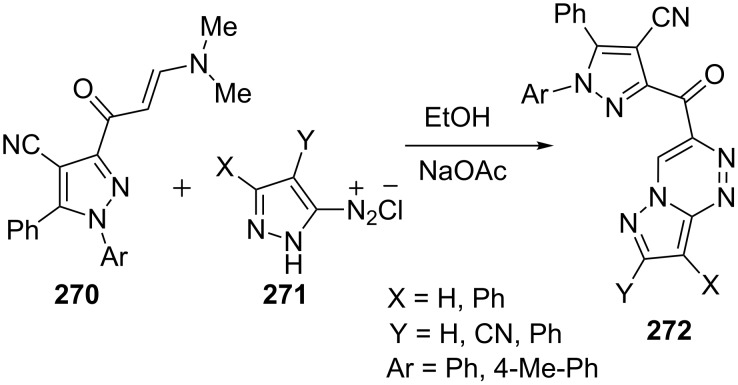
Synthesis of pyrazolo[5,1-*c*][1,2,4]triazines in presence of sodium acetate.

Abdelhamid et al. [165] in a similar report synthesized pyrazolo[5,1-*c*][1,2,4]triazines **272** from the reaction of enaminone **270** with pyrazol-3-yl diazonium salt **271** in ethanolic sodium acetate solution (Scheme 74).

**Scheme 75:**
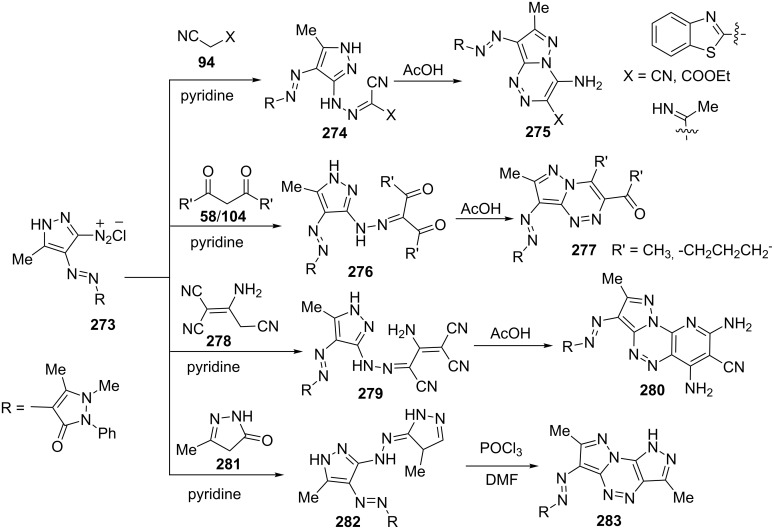
Synthesis of various 7-diazopyrazolo[5,1-*c*][1,2,4]triazine derivatives.

Metwally et al. [166] reported the synthesis of pyrazolo[5,1-*c*][1,2,4]triazines **275**, **277**, **280** and **283** from coupling of pyrazolyldiazonium salt **273** with various nitrile derivatives **94**, β-diketones **58**, 2-aminoprop-1-ene-1,1,3-tricarbonitrile (**278**) and 3-methyl-1*H*-pyrazol-5(4*H*)-one (**281**) involving the intermediacy of corresponding hydrazones **274**, **276**, **279** and **282** in acetic acid or POCl_3_/DMF (Scheme 75).

**Scheme 76:**
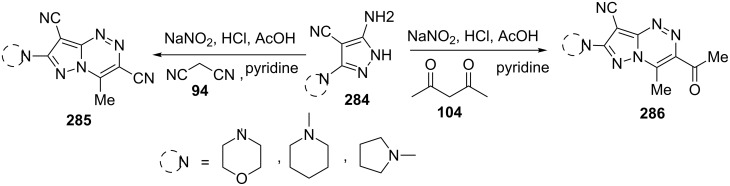
One pot synthesis of pyrazolo[5,1-*c*][1,2,4]triazines.

Adapting a similar procedure Al-Adiwish et al. [167] reported the synthesis of pyrazolo[5,1-*c*][1,2,4]triazines **285** and **286**. 5-Aminopyrazoles **284** were diazotized to the corresponding diazonium salts and subsequently condensed with active methylene **94** and **104** to give hydrazono intermediates which underwent cyclization in acetic acid to provide the desired pyrazolo[5,1-*c*][1,2,4]triazines **285** and **286,** respectively (Scheme 76). Selected pyrazolo[5,1-*c*][1,2,4]triazines **285** and **286** were screened for antibacterial activity and cytotoxicity against Vero cells.

**Scheme 77:**
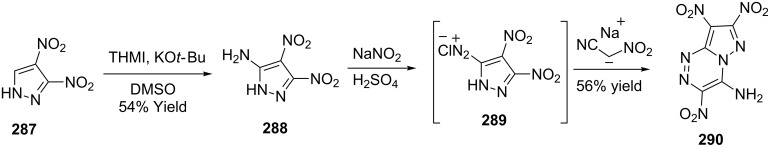
Synthesis of 4-amino-3,7,8-trinitropyrazolo-[5,1-*c*][1,2,4]triazines.

Schulze et al. [168] reported that 3,4-dinitropyrazole (**287**) on treatment with trimethylhydrazinium iodide (TMHI) provided 5-amino-3,4-dinitropyrazole (**288**) in 54% yields. The subsequent diazotization of aminopyrazole **288** and its coupling with sodium salt of nitroacetonitrile provided with 56% of 4-amino-3,7,8-trinitropyrazolo-[5,1-*c*][1,2,4]triazine (**290**) which has promising explosive properties (Scheme 77).

**Scheme 78:**
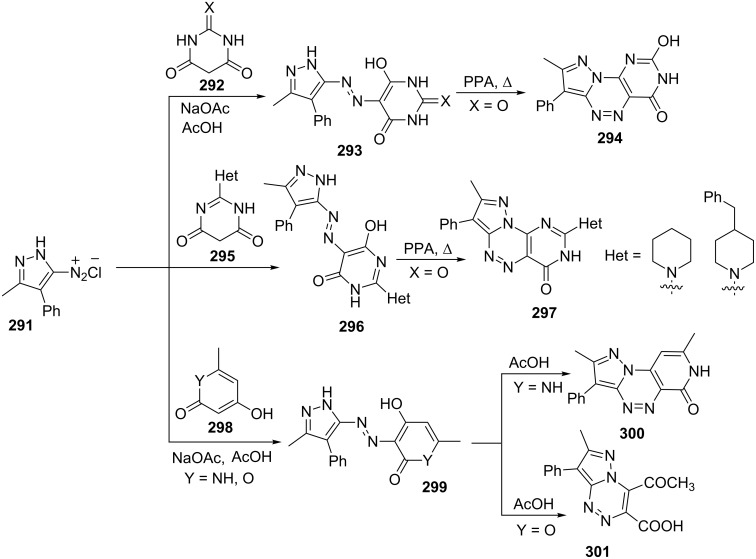
Synthesis of tricyclic pyrazolo[5,1-*c*][1,2,4]triazines by azocoupling reaction.

Ledenyova et al. [169] have reported the synthesis of tricyclic pyrazolo[5,1-*c*][1,2,4]triazines **294**, **297**, **300** and **301** from azocoupling reaction of pyrazolediazonium salts **291** with various heterocyclic components, e.g., barbituric acid and thiobarbituric acid **292**. Attempts were made to cyclize azocoupled intermediates **293** by heating with polyphosphoric acid (PPA) but only the intermediate formed from barbituric acid (X = O) provided pyrazolo[5,1-*c*]pyrimido[4,5-*e*][1,2,4]triazine-4(3*H*)-one **294** while the intermediate (X = S) failed to cyclize with PPA and anhydrous sodium acetate in acetic acid (Scheme 78).

The reaction of pyrazolediazonium salts **291** with 2-hetarylpyrimidine-4,6-diones **295** in the presence of sodium acetate in acetic provided coloured compounds **296** which underwent smooth cyclization on heating with PPA to give 8-methyl-7-phenyl-2-hetaryl-pyrazolo[5,1-*c*]pyrimido[4,5-*e*][1,2,4]triazin-4(3*H*)-ones **297** in 65–70% yields.

The coupling reaction of 4-hydroxy-6-methyl-1*H*-pyridine-2-one 4-hydroxy-6-methyl-2*H*-pyran-2-one (triacetic acid, **298**) with pyrazolediazonium salts **291** provided pyrazolo[5,1-*c*][1,2,4]triazine derivatives **300** through the intermediacy of azo coupled product **299**. While 4-hydroxy-6-methyl-2*H*-pyran-2-one (**298**, Y = O) underwent cyclization differently with formation of bicyclic carboxylic acid derivative of pyrazolo[5,1-*c*][1,2,4]triazine **301** probably due to lactone ring opening (Scheme 78).

## Conclusion

In this review article, we have systematically summarized various synthetic methods developed in the last decade for the construction of various pyrazoloazines as a group of fused pyrazolo derivatives utilizing 5-aminopyrazole as a synthetic precursor. The 5-aminopyrazole nucleus possesses ubiquitous distinctive structural features and its coupling reactions with different types of electrophilic reagents like aldehydes, ketones, β-diketones, β-ketoesters, and α,β-unsaturated ketones truly justifies its synthetic potential to construct fused heterocycles. This review opens the scope for future developments in new methodologies which promise the synthesis of novel fused heterocyclic systems with a wide range of medicinal and synthetic applications.
